# Targeting Cannabinoid 1 Receptor in Macrophages Alleviates Psoriatic Skin Inflammation Through the AUF1–NLRP3 Axis

**DOI:** 10.1002/advs.78313

**Published:** 2026-10-14

**Authors:** Hongseo Choi, Sukyoung Han, Jinho Kim, Ji Hye Han, Eunsu Kim, Yeonjeong Heo, Minju Lee, Jooyoung Lee, Heerim You, Soyoun Lee, Su‐Hee Han, Ilrok Yoon, Sung Anjeon, Hyerim Kang, Seungyeon Ryu, Hyun‐Ji Park, Eunha Kim, Chul Hwan Bang, Ji Hyun Lee, Josephine M. Egan, Jung‐Woong Kim, Eun Kyung Lee, Wook Kim

**Affiliations:** ^1^ Department of Molecular Science & Technology Ajou University Suwon Republic of Korea; ^2^ Department of Medical Science, Graduate School The Catholic University of Korea Seoul Republic of Korea; ^3^ Department of Life Science Chung‐Ang University Seoul Republic of Korea; ^4^ Advanced College of Bio‐Convergence Engineering Ajou University Suwon Republic of Korea; ^5^ Department of Dermatology, Seoul St. Mary's Hospital, College of Medicine The Catholic University of Korea Seoul Republic of Korea; ^6^ National Institute on Aging National Institutes of Health Baltimore Maryland USA; ^7^ Department of Biochemistry, College of Medicine The Catholic University of Korea Seoul Republic of Korea

**Keywords:** AUF1, cannabinoid 1 receptor (CB1R), immunometabolism, macrophage, NLRP3, psoriasis, skin inflammation

## Abstract

Psoriasis is a chronic inflammatory skin disease associated with systemic metabolic dysregulation; however, the molecular pathways linking metabolic signaling to skin inflammation remain poorly understood. Here, we identify cannabinoid 1 receptor (CB1R) in macrophages as a key immunometabolic regulator of psoriatic inflammation and a potential therapeutic target. CB1R expression was increased in macrophages infiltrating psoriatic lesional skin in both humans and mice, and both pharmacological inhibition and genetic deletion of CB1R markedly attenuated psoriasis‐like pathology in mouse models by reducing epidermal hyperplasia, inflammation, and barrier dysfunction in a macrophage‐dependent manner. Mechanistically, CB1R signaling in macrophages suppressed expression of the RNA‐binding protein AUF1, leading to stabilization of *Nlrp3* mRNA and enhanced NLRP3 inflammasome activation. AUF1 directly bound an AU‐rich region within the *Nlrp3* 3′ untranslated region and promoted mRNA destabilization, thereby limiting inflammasome activation and IL‐1β production. Restoration of AUF1 expression, either by CB1R inhibition or overexpression, reduced NLRP3 levels and alleviated psoriasis‐like pathology, whereas *Nlrp3* deficiency abrogated these effects. Collectively, these findings define a CB1R–AUF1–NLRP3 signaling axis that links metabolic signaling to inflammasome‐driven skin inflammation in macrophages. Targeting macrophage CB1R may therefore represent a promising therapeutic strategy for psoriasis and other immunometabolic inflammatory diseases.

## Graphical abstract

Macrophage CB1R emerges as a key driver of psoriatic inflammation by suppressing AUF1‐mediated post‐transcriptional control of NLRP3. CB1R inhibition restores AUF1‐dependent NLRP3 mRNA decay, restrains inflammasome activation, and alleviates psoriasis‐like inflammation. These findings establish a CB1R–AUF1–NLRP3 immunometabolic axis and highlight macrophage CB1R as a potential therapeutic target for psoriasis.

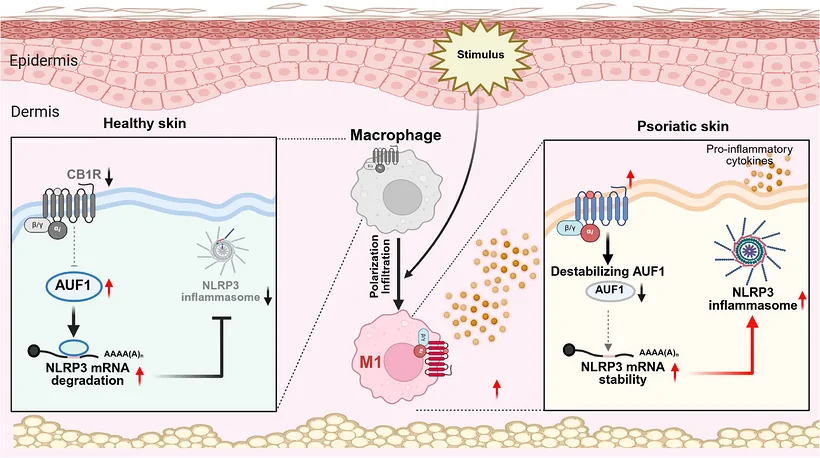

## 1 Introduction

Psoriasis is a chronic immune‐mediated skin disease characterized by recurrent erythematous plaques with scaling, epidermal hyperplasia, impaired keratinocyte differentiation, and extensive immune‐cell infiltration. At the molecular level, psoriasis is driven by the IL‐23/IL‐17 axis, in which dysregulated crosstalk between immune cells and keratinocytes promotes excessive production of pro‐inflammatory cytokines, including IL‐1β, IL‐17A, IL‐23, and TNF‐α [1, 2, 3, 4]. Biologic therapies targeting these cytokines—including anti‐IL‐17, anti‐IL‐23, and anti‐TNF‐α agents—have substantially improved disease management. However, many patients relapse after treatment discontinuation, and prolonged systemic immunosuppression may increase the risk of infection [3, 4, 5, 6, 7]. Identifying upstream regulatory mechanisms that sustain psoriatic inflammation therefore remains an important goal for developing more durable therapeutic strategies.

Beyond the skin, psoriasis is increasingly recognized as a systemic inflammatory disease associated with metabolic disorders, including obesity, type 2 diabetes (T2D), and cardiovascular disease [4, 5, 6, 8, 9, 10]. These clinical associations suggest shared immunometabolic pathways linking metabolic stress to cutaneous inflammation; however, the molecular basis of this connection remains poorly defined. Cannabinoid 1 receptor (CB1R), a G protein‐coupled receptor that regulates energy balance, glucose metabolism, and lipid signaling, represents a potential mechanistic link between metabolic and inflammatory processes [11, 12, 13, 14]. Pharmacological CB1R antagonism with first‐generation compounds such as rimonabant (RIMO) showed potent metabolic and anti‐inflammatory effects but was discontinued clinically due to neuropsychiatric adverse effects associated with central CB1R inhibition [15, 16, 17, 18, 19]. This limitation has prompted the development of second‐generation peripherally restricted CB1R antagonists designed to limit brain exposure while retaining peripheral efficacy, with potential applications in chronic inflammatory and fibrotic disorders, including liver and kidney fibrosis [19, 20, 21, 22, 23, 24]. Notably, endocannabinoid (EC)‐mediated CB1R activation in macrophages within metabolic tissues can promote inflammasome‐dependent inflammation [25, 26]. Despite increasing interest in EC signaling in inflammatory diseases, whether CB1R contributes to inflammatory skin disease—particularly macrophage‐driven psoriasis—and how it regulates inflammasome activation remain unclear. Furthermore, limited available studies have reported conflicting pro‐ and anti‐inflammatory effects of CB1R depending on the cellular context [27, 28, 29], leaving the functional role of CB1R in skin inflammation unresolved.

Post‐transcriptional regulation of inflammatory gene expression is increasingly recognized as a critical mechanism shaping macrophage inflammatory programs. RNA‐binding proteins (RBPs) control the stability, localization, and translation of transcripts encoding inflammatory mediators, thereby enabling macrophages to rapidly adapt to dynamic inflammatory cues [30, 31, 32]. Among these regulators, AU‐rich element RNA‐binding protein 1 (AUF1, also known as hnRNP D) functions as an mRNA‐destabilizing factor that promotes the degradation of multiple pro‐inflammatory transcripts, including those encoding IL‐6, TNF‐α, iNOS, and GM‐CSF [33, 34, 35, 36, 37]. Consistent with this regulatory role, AUF1 deficiency results in systemic hyperinflammation resembling autoimmune disease [37, 38]. Although RBPs including AUF1 regulate the stability and translation of inflammatory transcripts, how inflammasome components are regulated post‐transcriptionally remains poorly understood. In particular, whether AUF1 directly regulates mRNAs encoding inflammasome components during macrophage‐mediated skin inflammation has not been defined.

Here we investigated whether macrophage CB1R promotes psoriatic inflammation through AUF1‐dependent post‐transcriptional regulation of the NLRP3 inflammasome. We show that CB1R activation suppresses AUF1 expression, thereby stabilizing *Nlrp3* mRNA and enhancing inflammasome‐dependent IL‐1β production. Conversely, pharmacological or genetic inhibition of CB1R restores AUF1 expression, destabilizes *Nlrp3* mRNA, and alleviates psoriatic inflammation. We further demonstrate that AUF1 directly binds to an AU‐rich region (AU) within the 3′ untranslated region (3′UTR) of *Nlrp3* mRNA, promoting transcript destabilization and limiting inflammasome activation. Together, these findings identify AUF1 as a post‐transcriptional checkpoint of NLRP3 expression and establish the CB1R–AUF1–NLRP3 axis as a previously unrecognized regulatory pathway linking EC signaling to RNA‐mediated control of innate immune activation in psoriasis.

## 2 Results

### 2.1 CB1R Expression Is Elevated in Psoriatic Skin and Enriched in Infiltrating Macrophages

CB1R (gene *Cnr1*) has been implicated in macrophage‐driven inflammation in metabolic tissues such as adipose tissue [39, 40], liver [41, 42, 43], and pancreatic islets [19, 25, 26], and EC signaling activates inflammasome programs in infiltrating macrophages in metabolic disease [19, 25, 26]. In contrast, the expression and function of macrophage CB1R in inflammatory skin diseases have remained largely undefined. Thus, we first examined CB1R expression in psoriatic mouse skin. CB1R protein abundance was significantly higher in the dorsal skin of imiquimod (IMQ)‐inflamed mice and the ear skin of rIL‐23‐inflamed mice than in the corresponding control tissues (Figure 1A). To determine whether increased CB1R expression is associated with disease severity, we next examined the temporal relationship between CB1R abundance and the progression of IMQ‐induced psoriasis‐like inflammation. Following daily IMQ application, psoriasis area and severity index (PASI) scores increased over time, accompanied by splenomegaly and a gradual increase in epidermal thickness (Figure S1A–D). Consistent with these clinical and histopathological changes, cutaneous levels of the psoriasis‐associated inflammatory cytokines TNF‐α, IL‐1β, IL‐17A, and IL‐23p19 also increased during disease progression (Figure S1E). Notably, CB1R protein abundance in the dorsal skin showed a concomitant increase over the same period (Figure S1F), closely paralleling the increases in clinical severity, epidermal hyperplasia, and cutaneous inflammation. These findings indicate that increased cutaneous CB1R expression is associated with the progression and severity of IMQ‐induced psoriasis‐like inflammation. Next, to verify macrophage CB1R expression in psoriatic skin, we generated a 6‐carboxyfluorescein (6‐FAM)‐conjugated anti‐CB1R antibody (CB1R‐FAM) and established whole‐body (*Cnr1* ^−/−^; CMV‐Cre × *Cnr1* ^flox/flox^) and myeloid‐specific *Cnr1* knockout mice (M‐*Cnr1* ^−/−^; LysM‐Cre × *Cnr1* ^flox/flox^). In mouse primary bone marrow‐derived macrophages (BMDMs), CB1R‐FAM bound robustly to wild‐type (WT) cells with a much‐reduced signal in *Cnr1* ^−/−^ and M‐*Cnr1* ^−/−^ BMDMs, as assessed by flow cytometry (Figure S2A,B) and confocal microscopy (Figure S2C), confirming probe specificity. Knockout genotypes were verified by genomic PCR (Figure S3A,B) and by confirming the loss of *Cnr1* mRNA and protein in the brain, BMDMs, and bone marrow‐derived dendritic cells (BMDCs) (Figure S3C–E). Consistent with macrophage‐specific deletion, CB1R expression was preserved in the brain and BMDCs of WT and M‐*Cnr1* ^−/−^ but not *Cnr1* ^−/−^ mice (Figure S3C–E). In contrast, CB1R expression in BMDMs was detected only in WT mice and was absent in both *Cnr1* ^−/−^ and M‐*Cnr1* ^−/−^ mice (Figure S3C,E). Baseline immune‐cell profiles were comparable across genotypes (Figure S4). Flow cytometry of skin single‐cell suspensions using CB1R‐FAM revealed an increased fraction of CB1R^+^ cells within CD45^+^CD11b^+^F4/80^+^ macrophages in IMQ‐ and rIL‐23‐inflamed skins compared with control (Figure 1B; Figures S5 and S6). Confocal imaging showed CB1R co‐localization with macrophage markers (CD68 and F4/80) in lesional dermis of IMQ‐ and rIL‐23‐inflamed WT mice, concomitant with increased macrophage infiltration [44, 45], but no CB1R signals in lesional dermis of IMQ‐ and rIL‐23‐inflamed *Cnr1* ^−/−^ mice (Figure 1C; Figure S7). To define the macrophage subtype in which CB1R is induced, BMDMs were exposed to M1‐polarizing stimuli (LPS or IMQ; CD86^+^), which significantly increased CB1R expression (Figure 1D,E), whereas M2‐polarizing conditions (IL‐4; CD206^+^) had no effect (Figure 1E). In keeping with the murine data, analysis of human RNA‐seq cohorts revealed higher *CNR1* mRNA in psoriatic lesions than in non‐lesional or healthy skin (Figure 1F). Consistent with the transcriptomic data, immunofluorescence analysis of human skin tissues revealed increased CB1R staining in CD68^+^ macrophages within psoriatic lesions compared with normal skin. Quantitative analysis further demonstrated a significantly higher proportion of CB1R^+^CD68^+^ cells among dermal CD68^+^ macrophages in psoriatic lesional skin than in normal skin (Figure 1G). Together, these findings demonstrate increased macrophage‐associated CB1R expression in both mouse and human psoriatic skin.

**FIGURE 1:**
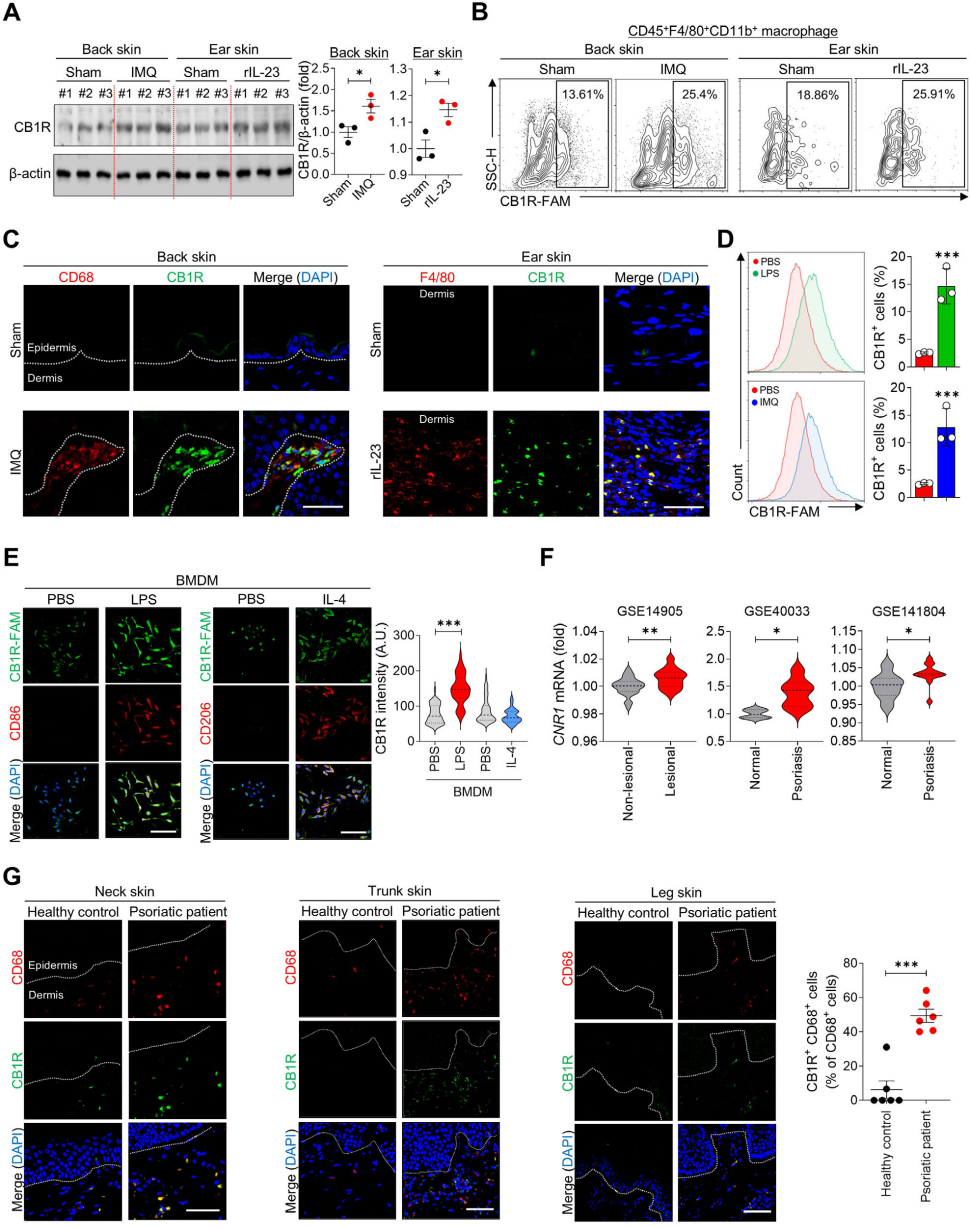
Macrophage CB1R expression is increased in psoriatic skin lesions in mice and humans. (A) Western blots of CB1R in dorsal skin from IMQ‐inflamed mice and ear skin from rIL‐23‐inflamed mice; β‐actin, loading control. Densitometric quantification of CB1R normalized to β‐actin is shown at right (*n*  =  3 mice per group). (B) Flow‐cytometric analysis of skin single‐cell suspensions. Representative gating plots depict the frequency of CB1R^+^ cells within CD45^+^F4/80^+^CD11b^+^ macrophages from dorsal and ear skins of control and IMQ‐ or rIL‐23‐inflamed mice. Cells from five mice per group were pooled for analysis. (C) Confocal immunofluorescence of dorsal and ear skin sections showing CB1R (green), macrophage markers (CD68 or F4/80, red), and nuclei (DAPI, blue) in control and IMQ‐ or rIL‐23‐inflamed mice. Scale bars, 50 µm. (D) Flow‐cytometric analysis and quantification of CB1R‐FAM binding in F4/80^+^CD11b^+^ BMDMs after stimulation with LPS (top) or IMQ (bottom) (*n* = 3 per group). (E) Confocal immunofluorescence of BMDMs polarized to M1 (LPS; CD86^+^) or M2 (IL‐4; CD206^+^). Cells were stained for CB1R (green) together with CD86 or CD206 (red). Quantification of CB1R fluorescence intensity is shown at right (*n* = 50 cells per group). Scale bars, 100 µm. (F) *CNR1* (gene encoding CB1R) mRNA expression in healthy control skin (*n* = 4–28) and psoriatic lesional skin (*n* = 7–34) from RNA‐seq datasets (No. GSE14905, GSE40033, and GSE141804). (G) Representative confocal immunofluorescence images of neck, trunk, and leg skin sections from healthy controls and patients with psoriasis showing CB1R (green), CD68 (red), and nuclei (DAPI, blue). Scale bars, 100 µm. Quantification of CB1R^+^CD68^+^ macrophages, expressed as a percentage of total CD68^+^ macrophages for each individual subject is shown on right for healthy controls (*n* = 6) and patients (*n* = 6) with psoriasis. Data are presented as mean ± SEM. * *p* < 0.05; ** *p* < 0.01; *** *p* < 0.001, analyzed by unpaired two‐tailed Student's *t* test (A, D, E, F, G).

### 2.2 Pharmacological CB1R Blockade Ameliorates IMQ‐Induced Psoriasis‐Like Phenotypes and Restores Epidermal Barrier Function

To assess the role of CB1R in psoriasis pathogenesis, we tested the first‐generation CB1R antagonist RIMO in the IMQ‐induced psoriasis mouse model. The RIMO used in this study was synthesized in‐house and exhibited high‐affinity CB1R binding (K_i_ = 4.4 nm) and potent functional activity in cAMP (IC_50_ = 13.8 nm) and β‐arrestin (IC_50_ = 2.1 nm) assays, with a brain‐to‐plasma ratio of 1.22 ± 0.21. These values were consistent with the previously reported pharmacological properties of RIMO [21, 23, 24, 46, 47, 48]. C57BL/6 mice received daily intraperitoneal RIMO (10 mg kg^−1^) 30 min before topical 5% IMQ for 4 consecutive days on shaved dorsal skin (Figure 2A). RIMO protected mice from IMQ‐induced psoriasis‐like phenotypes, including any increase of PASI scores, splenomegaly, and epidermal hyperplasia (Figure 2B–E). At the molecular level, RIMO reduced the IMQ‐induced mRNA expression of *Cnr1*, NLRP3 inflammasome component genes (*Nlrp3* and *Casp1*), and pro‐inflammatory genes (*Tnf*, *Il1b*, *Il6*, and *Ptgs2*) (Figure 2F,G; Figure S8A–C), accompanied by decreased TNF‐α and IL‐1β protein levels (Figure S8D). In CD45^+^ immune cells isolated from dorsal skin (Figure S9), IMQ‐induced increases in *Tnf*, *Il1b*, and the macrophage marker *Cd68* mRNAs were suppressed by RIMO (Figure 2H). Flow cytometry of immune cell populations from dorsal skin also showed that RIMO reversed IMQ‐induced infiltration of CD45^+^ immune cells, CD45^+^CD3^+^ T cells, CD45^+^Ly6G^+^ neutrophils, and CD45^+^F4/80^+^CD11b^+^CD86^+^ M1 macrophages (Figure 2I; Figure S9). Comparable preventive effects were observed with another first‐generation antagonist AM251 and with peripherally restricted second‐generation CB1R antagonists (AM6545, JD5037, TM38837 and BPRCB1184), all of which reduced PASI scores, epidermal hyperplasia, and skin inflammation (Figure S10A–E).

**FIGURE 2:**
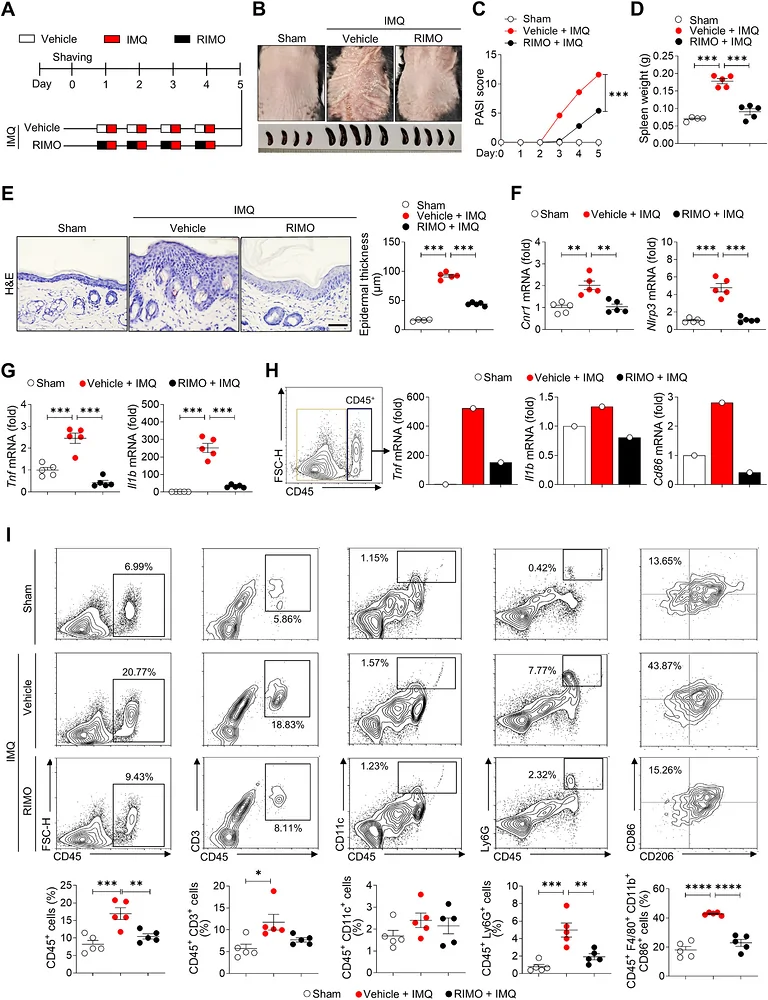
The first‐generation CB1R antagonist RIMO exerts anti‐psoriatic effects in the IMQ‐induced psoriasis mouse model. (A) Experimental design. RIMO (10 mg kg^−1^) or vehicle was administered intraperitoneally 30 min prior to topical application of Aldara cream (5% IMQ) on days 1 through 4 using a preventive treatment protocol. (B) Representative gross images of shaved dorsal skin (top) and spleens (bottom) on day 5. (C) Clinical PASI score of dorsal skin over 5 d for the mice shown in (B, top) (*n*  =  4–5 mice per group). (D) Spleen weight for the mice shown in (B, bottom) (*n*  =  4–5 per group). (E) Representative H&E images (left) and epidermal thickness (right) of dorsal skin sections on day 5 (*n*  =  4–5 mice per group). Scale bars, 100 µm. (F) *Cnr1* and *Nlrp3* mRNA levels in dorsal skin from each group (*n*  =  5 mice per group), determined by qPCR and normalized to *Gapdh*. (G) *Tnf* and *Il1b* mRNA levels in dorsal skin from the groups in (F), determined by qPCR and normalized to *Gapdh*. (H) *Tnf*, *Il1b*, and *Cd86* mRNA levels in CD45^+^ cells isolated from dorsal skins of each group. Cells from five mice per group were pooled, flow‐sorted, and analyzed by qPCR; values were normalized to *Gapdh*. (I) Representative flow cytometry plots and quantification of immune‐cell subsets in dorsal skins from each group. Live singlets in single‐cell suspensions were gated, and the frequencies of CD45^+^ immune cells, CD45^+^CD3^+^ T cells, CD45^+^CD11c^+^ dendritic cells, CD45^+^Ly6G^+^ neutrophils, and CD45^+^F4/80^+^CD11b^+^CD86^+^ macrophages were quantified (*n* = 5 mice per group). Data are presented as mean ± SEM. * *p* < 0.05; ** *p* < 0.01; *** *p* < 0.001; ****p<0.0001, analyzed by one‐way ANOVA with Tukey's multiple comparisons test (D−G, I) or two‐way ANOVA with Bonferroni's post hoc test (C).

Given that poor keratinocyte differentiation and defective barrier function are hallmarks of psoriasis [49, 50, 51], we next examined the effect of RIMO on skin barrier dysfunction of IMQ‐inflamed mice. RIMO treatment restored the lesional loricrin (gene *Lor*) and involucrin (gene *Ivl*) expression (Figure S11A)—terminal differentiation markers of epidermal keratinocytes [52]—an effect also observed in dorsal skin of IMQ‐inflamed *Cnr1* ^−/−^ and M‐*Cnr1* ^−/−^ mice (Figure S11B). Consistent with these findings, analysis of CD45^−^ non‐immune cells isolated from dorsal skin showed that RIMO reversed IMQ‐induced mRNA expression of *Lor*, antimicrobial peptide genes (*Defb3* and *S100a9*), and *Il6* (Figure S11C). At the protein level, western blot analysis showed that IMQ‐induced changes in barrier‐related proteins were normalized by RIMO: claudin‐1, a tight‐junction component [49, 53], was restored and S100A9 was reduced (Figure S11D), coincident with decreased transcutaneous flux of FITC–dextran in RIMO‐treated mice (Figure S11E). Together, these results demonstrate that pharmacological CB1R blockade prevents IMQ‐induced psoriasis‐like dermatitis and improves barrier integrity.

We next evaluated the therapeutic potential of RIMO in a delayed‐treatment setting. C57BL/6 mice received topical 5% IMQ for 4 d and were administered RIMO intraperitoneally twice beginning on day 3 (Figure S12A). Similar to the preventive setting (Figure 2; Figure S10), RIMO significantly alleviated IMQ‐induced disease phenotypes—reducing PASI scores, splenomegaly, epidermal hyperplasia, proinflammatory cytokine expression, and immune‐cell infiltration, including M1 macrophages (Figure S9; Figure S12B–G)—supporting CB1R as a promising therapeutic target for psoriasis.

### 2.3 Macrophages Are Required for the Anti‐Psoriatic Effect of RIMO

As shown in Figure 1, CB1R expression is closely associated with dermal macrophages in psoriatic lesions. They are central immune regulators in psoriasis pathogenesis, with their infiltration and activation contributing to hallmark features in humans and mice [44, 45, 54, 55, 56]. To directly test macrophage involvement in the efficacy of CB1R blockade, we used clodronate liposomes to deplete macrophages in vivo [44, 45, 54, 55, 56, 57]. Mice received daily RIMO (10 mg kg^−1^) via the intraperitoneal route for 4 d, 30 min prior to topical 5% IMQ, while clodronate or control liposomes were administered intraperitoneally on days 0 and 3 (Figure 3A). Macrophage depletion was confirmed by flow cytometry of peritoneal macrophages and anti‐CD68 immunohistochemistry in skin (Figure S13A,B). Compared with control liposomes, clodronate liposomes protected animals against IMQ‐induced increase in PASI scores, splenomegaly, epidermal hyperplasia, and skin inflammation (Figure 3B–E; Figure S13B,C). Notably, clodronate‐mediated macrophage depletion substantially diminished the protective effects of RIMO against IMQ‐induced disease phenotypes (Figure 3B–E; Figure S13B,C), indicating that macrophages contribute substantially to the anti‐psoriatic activity of RIMO. To further define the macrophage‐intrinsic contribution of CB1R to psoriasis‐like phenotypes, we performed macrophage reconstitution experiments following clodronate‐mediated macrophage depletion. Macrophage‐depleted mice were adoptively transferred with Far Red‐labeled WT or *Cnr1* ^−/−^ BMDMs before induction of psoriasis‐like dermatitis with IMQ (Figure S14A). Fluorescence imaging confirmed the presence of adoptively transferred WT and *Cnr1* ^−/−^ BMDMs in the dorsal skin (Figure S14B). As expected, clodronate‐mediated macrophage depletion markedly attenuated IMQ‐induced psoriasis‐like phenotypes, as reflected by reduced gross skin lesions, PASI scores, and epidermal hyperplasia (Figure S14C–E). Importantly, adoptive transfer of WT BMDMs significantly restored these disease phenotypes in macrophage‐depleted mice, whereas transfer of *Cnr1* ^−/−^ BMDMs failed to do so (Figure S14C–E). Consistent with these histopathological changes, adoptive transfer of WT BMDMs restored the IMQ‐induced increases in cutaneous TNF‐α and IL‐1β expression in macrophage‐depleted mice, whereas transfer of *Cnr1* ^−/−^ BMDMs failed to restore these inflammatory responses (Figure S14F). These findings provide direct genetic reconstitution evidence that macrophage CB1R is required for the pro‐inflammatory activity of macrophages during IMQ‐induced psoriasis‐like dermatitis.

**FIGURE 3:**
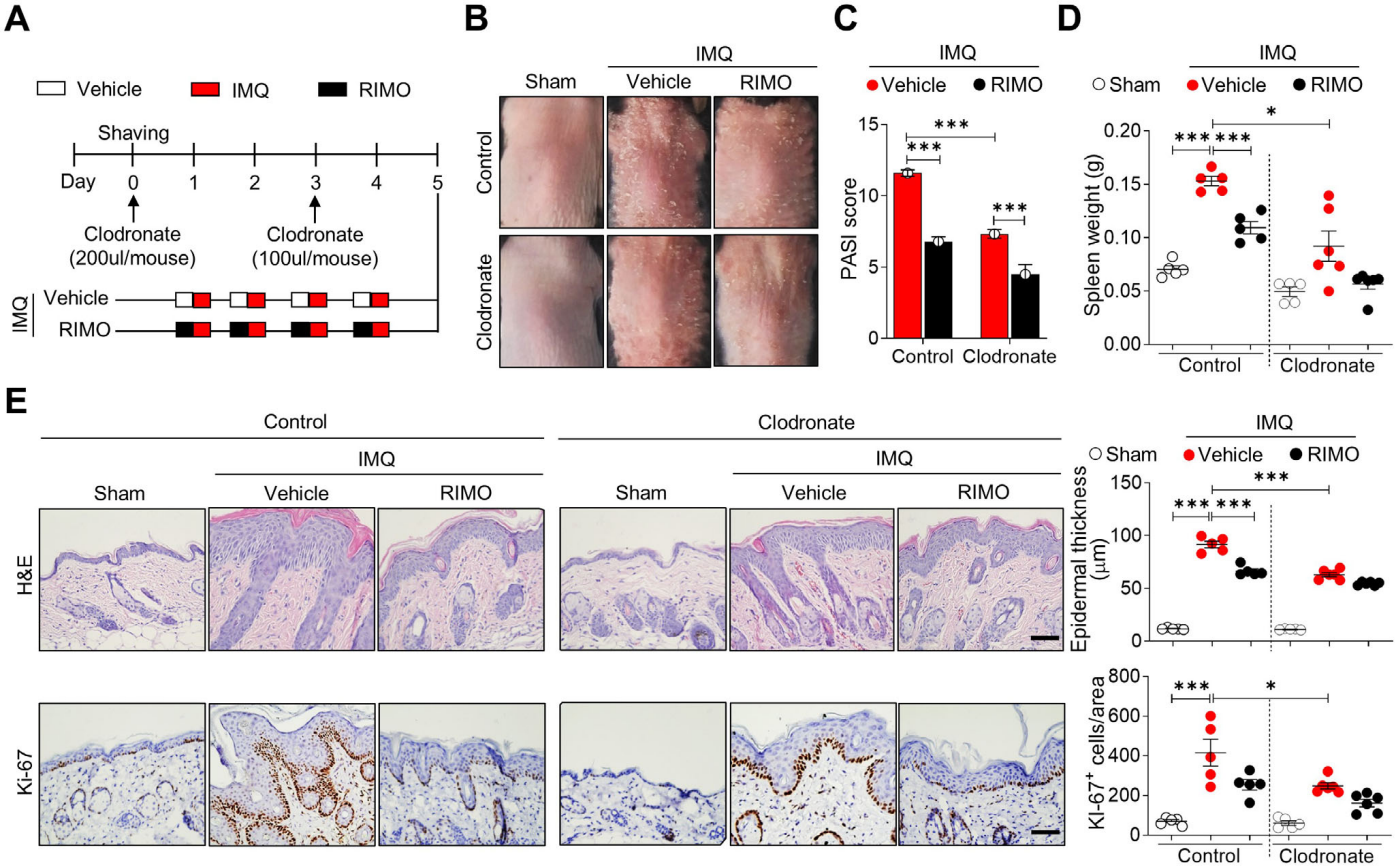
Depletion of macrophages diminishes the anti‐psoriatic effects of RIMO in vivo. (A) Experimental design. Control or clodronate liposomes (50 mg) were administered intraperitoneally on days 0 and 3. RIMO (10 mg kg^−1^) or vehicle was given intraperitoneally 30 min prior to topical application of Aldara cream (5% IMQ) on days 1–4 using a preventive treatment protocol. (B) Representative gross images of shaved dorsal skin on day 5. (C) Clinical PASI score of dorsal skin over 5 d for the mice (B, top) (*n*  =  5–6 mice per group). (D) Spleen weight for the mice (B, bottom) (*n*  =  5–6 per group). (E) Representative H&E images (top) and Ki‐67 immunohistochemistry (bottom) of dorsal skin sections on day 5. Epidermal thickness and the percentage of Ki‐67‐positive epidermal cells are shown at right (*n*  =  5 mice per group). Scale bars, 100 µm. Data are presented as mean ± SEM. * *p* < 0.05; ** *p* < 0.01; *** *p* < 0.001, analyzed by two‐way ANOVA followed by Tukey's multiple comparisons test (D, E) or Bonferroni's *post hoc* test (C).

### 2.4 Myeloid CellSpecific CB1R Deficiency Attenuates Psoriasis‐Like Inflammation

To further establish the contribution of macrophage CB1R to psoriasis‐like inflammation in vivo, we generated mice bearing a loxP‐flanked *Cnr1* exon 2 and crossed them with CMV‐Cre or LysM‐Cre lines to obtain *Cnr1* ^−/−^ and M‐*Cnr1* ^−/−^ mice, respectively (Figure S3A–E). WT, *Cnr1* ^−/−^, and M‐*Cnr1* ^−/−^ mice received topical 5% IMQ on shaved dorsal skin for 5 d. Compared with WT mice, both *Cnr1* ^−/−^ and M‐*Cnr1* ^−/−^ mice showed significant improvement in psoriasis‐like phenotypes, including PASI scores, splenomegaly, epidermal hyperplasia, pro‐inflammatory cytokine expression, and macrophage infiltration (Figure 4A–E; Figure S15). Although the improvements tended to be slightly greater in *Cnr1* ^−/−^ mice, the degree of amelioration in M‐*Cnr1* ^−/−^ mice was comparable, suggesting a dominant role for myeloid CB1R. The protective effects of CB1R deficiency were further confirmed in an rIL‐23‐induced model: daily intradermal rIL‐23 (500 ng per mouse) injection for 8 d provoked splenomegaly, epidermal hyperplasia, pro‐inflammatory cytokine expression, and macrophage infiltration in ear skin of WT mice, all of which were markedly attenuated in both *Cnr1* ^−/−^ and M‐*Cnr1* ^−/−^ mice (Figure S16A–D). In parallel, RIMO (10 mg kg^−1^) given 30 min prior to intradermal rIL‐23 reduced dermal infiltration of CD45^+^ immune cells, CD45^+^CD3^+^ T cells, CD45^+^CD11c^+^ dendritic cells, CD45^+^Ly6G^+^ neutrophils, and CD45^+^F4/80^+^CD11b^+^ macrophages in ear skin of WT mice (Figure S9; Figure S17).

**FIGURE 4:**
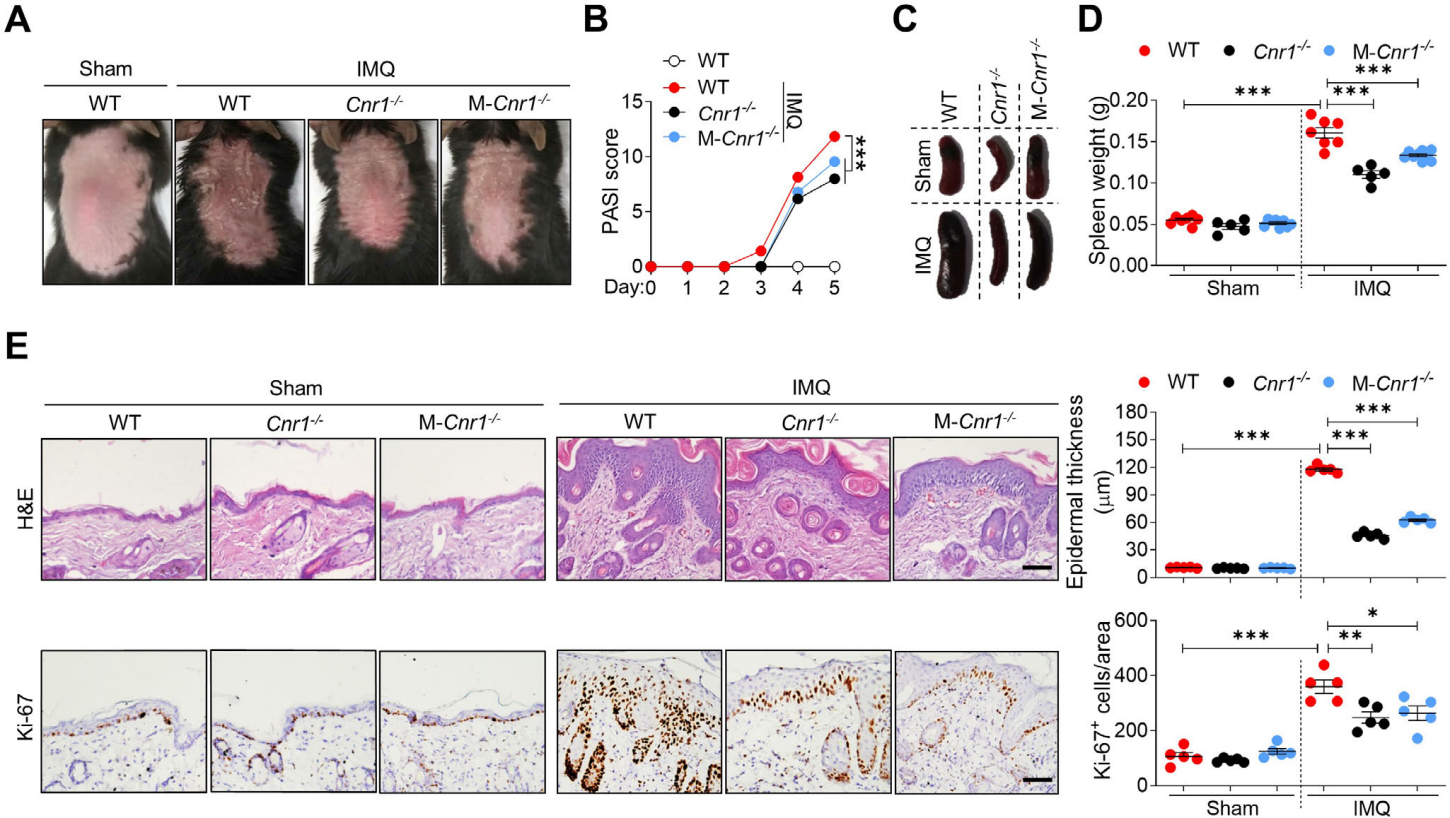
Myeloid cell‐specific CB1R deficiency ameliorates IMQ‐induced psoriasis‐like phenotypes in vivo. (A) Representative gross images of shaved dorsal skin on day 5. WT, *Cnr1^−/−^*, and M‐*Cnr1^−/−^* mice received topical PBS or Aldara cream (5% imiquimod, IMQ) once daily on days 1–4. (B) Clinical PASI score of dorsal skin over 5 d for the mice shown in (A) (*n*  =  6–9 mice per group). (C) Representative spleen images of the mice shown in (A). (D) Spleen weight for the mice shown in (A) (*n*  =  5–9 per group). (E) Representative H&E images (top) and Ki‐67 immunohistochemistry (bottom) of dorsal skin sections on day 5. Epidermal thickness and the percentage of Ki‐67‐positive epidermal cells are shown at right (*n*  =  5–6 mice per group). Scale bars, 100 µm. Data are presented as mean ± SEM. * *p* < 0.05; ** *p* < 0.01; *** *p* < 0.001, analyzed by two‐way ANOVA with Tukey's multiple comparison test (B, D, E).

We next determined whether macrophage CB1R drives pro‐inflammatory M1 polarization. For this, we employed LPS and IMQ as a key driver of M1 polarization. In WT BMDMs, LPS stimulation markedly upregulated expression of genes (*Il1b, Tnf, Il6, Il23a*, and *Il12b*) characteristic of the M1 macrophage phenotype, which was significantly blunted in *Cnr1* ^−/−^ BMDMs (Figure S18A). LPS‐induced expression of these genes was also suppressed by RIMO in RAW264.7 macrophages (Figure S18B). Similarly, IMQ‐induced expression of pro‐inflammatory M1 marker genes was attenuated in *Cnr1* ^−/−^ BMDMs (Figure S18C). Flow cytometry confirmed that RIMO reduced F4/80^+^CD11b^+^CD86^+^ M1‐macrophage frequency in LPS‐stimulated WT BMDMs, while *Cnr1* deletion both attenuated LPS‐driven expansion of the CD86^+^ subset and abolished any additional suppression by RIMO (Figure S18D–F). In addition, IMQ‐induced increases in M1 macrophages were also inhibited by RIMO (Figure S18F). These results indicate that macrophage CB1R exacerbates psoriasis‐like skin dermatitis by promoting M1 polarization.

### 2.5 Immune Cell Transcriptomics Reveal Reversal of Disease Programs by CB1R Blockade and Nominate an AUF1–NLRP3 Axis

To elucidate how CB1R influences macrophage polarization at the molecular level, we carried out RNA‐seq of CD45^+^ immune cells sorted from dorsal skin of IMQ‐inflamed mice and ear skin of rIL23‐inflamed mice, with or without RIMO (10 mg kg^−1^). Principal component analysis (PCA) of the RNA‐seq dataset showed clear separation among normal control (Sham), psoriatic (IMQ or rIL‐23), and RIMO groups with tight replicate clustering (Figure 5A,B). Hierarchical clustering confirmed divergent global expression profiles and robust transcriptomic alterations in both models (Figure 5C,D). Differential expression analysis identified 1,032 differentially expressed genes (DEGs) in IMQ versus Sham and 1,623 DEGs in rIL23 versus Sham (*p* < 0.05, |log_2_FC| > 1) (Figure S19A,B). Comparisons with RIMO groups at a relaxed threshold (*p* < 0.05) identified 537 and 872 counter‐regulated genes in IMQ and rIL‐23 groups, respectively (Figure S19C,D). A subset of psoriatic DEGs was consistently reversed by RIMO (Figure 5E,F). Pathway enrichment analysis of up‐ and down‐regulated DEGs highlighted IMQ‐ or rIL‐23‐induced pathways attenuated by RIMO, prominently included “lipid and atherosclerosis,” “osteoclast differentiation,” “NOD‐like receptor (NLR) signaling,” “apoptosis,” and “efferocytosis” (Figure 5G), among which NLR signaling emerged as the most consistent signature. In contrast, pathways suppressed by IMQ/rIL23 but restored by RIMO were limited, with “Human T‐cell leukemia virus 1 infection” the only recurrent term (Figure S19E). Weighted gene co‐expression network analysis (WGCNA) identified 19 modules; the green module was strongly associated with psoriatic groups and significantly enriched for NOD‐like receptor signaling (*p* < 3.9 × 10^−^^5^), as well as other inflammatory pathways (Figure 5H). Integration with STRING‐based protein–protein interaction (PPI) data refined this module to a 47‐node/67‐edge subnetwork centered on NLRP3 and IL‐1β (Figure 5I), nominating the NLRP3 inflammasome axis as a transcriptional hub induced by psoriatic stimuli and counter‐regulated by RIMO.

**FIGURE 5:**
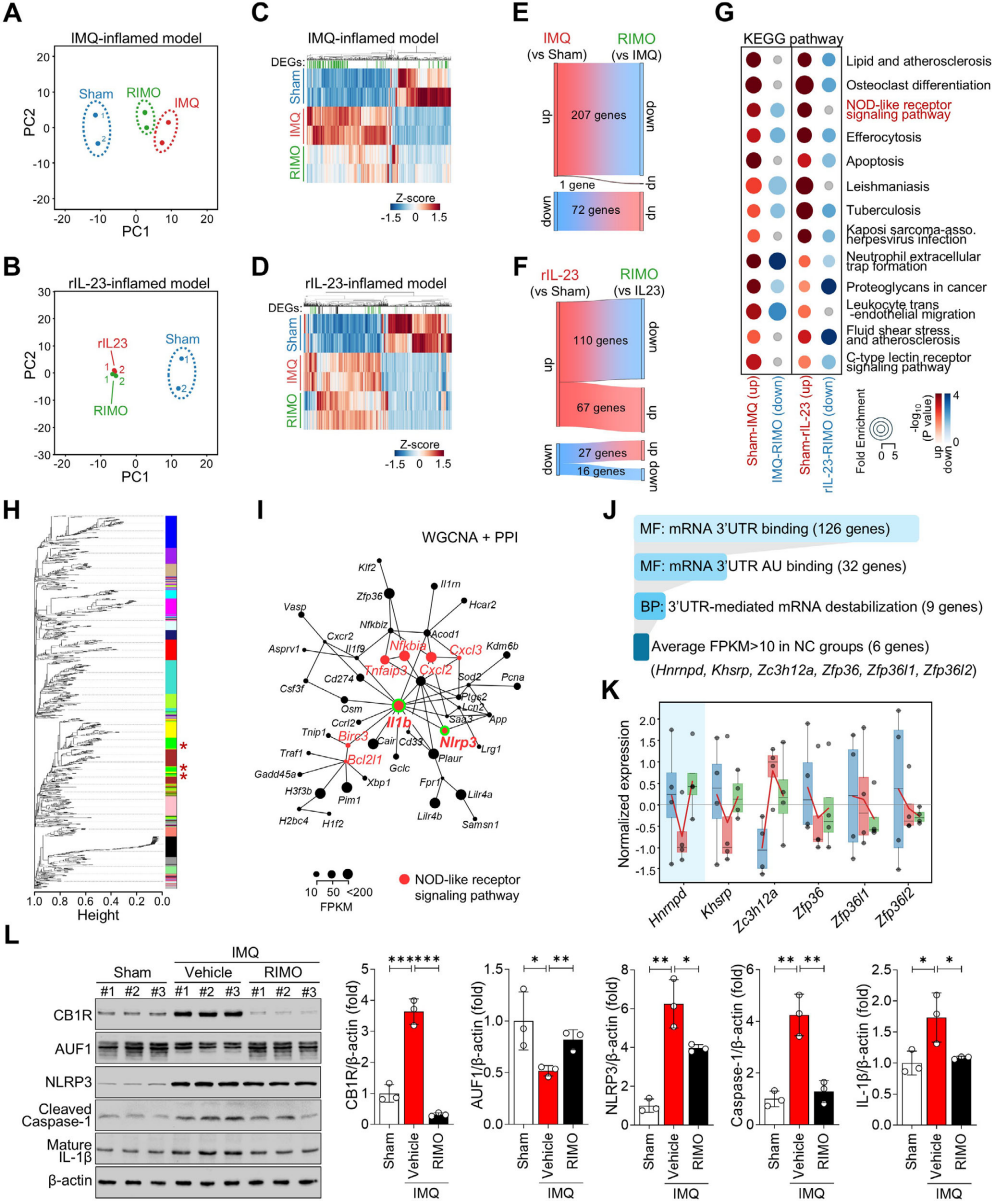
CB1R blockade reverses disease‐associated transcriptomic alterations and implicates an AUF1–NLRP3–IL‐1β regulatory axis in CD45^+^ immune cells from IMQ‐ and rIL‐23‐inflamed mice. (A, B) Principal component analysis (PCA) of RNA‐seq dataset from sorted CD45^+^ immune cells in dorsal skin of IMQ‐inflamed mice (A) and ear skin of rIL23‐inflamed mice (B) treated with vehicle or RIMO (10 mg kg^−1^). Groups are color‐coded as Sham (blue), IMQ or IL23 (red), and RIMO (green). (C, D) Hierarchical‐clustering heatmaps of differentially expressed genes (DEGs) from the dataset of (A) and (B). Annotation bars indicate DEGs reversed by RIMO (green) or concordant across groups (black). (E, F) Sankey diagrams classifying DEGs from (A) and (B) according to direction of change in disease and reversal by RIMO. (G) Bubble chart of KEGG pathway enrichment. Red dots indicate pathways upregulated in IMQ‐ or rIL‐23‐inflamed group, blue dots indicate downregulated pathways, and gray dots denote non‑significant terms; dot size reflects gene counts. (H) Weighted gene co‐expression network analysis (WGCNA) dendrogram showing module assignment (module colors indicated). (I) Integrated WGCNA–protein–protein interaction (PPI) network; co‐expression modules merged with curated PPIs. Nodes related to the NOD‐like receptor signaling pathway are highlighted in red. (J) Schematic illustrating candidate RNA‐binding proteins predicted to regulate *Nlrp3* mRNA via 3′UTR‐mediated destabilization. (K) Box‐and‐scatter plots of normalized expression for six candidate RNA‐binding protein genes across groups. Red lines denote group means; each black dot represents an individual sample. Box colors follow (A and B): NC (blue), IMQ/rIL‐23 (red), and RIMO (green). (L) Western blots of CB1R, AUF1, NLRP3, cleaved caspase‐1, and mature IL‐1β in dorsal skin from control and IMQ‐inflamed mice treated with vehicle or RIMO (10 mg kg^−1^); β‐actin, loading control. Densitometric quantification of CB1R normalized to β‐actin is shown at right (*n * =  3 mice per group). Data are presented as mean ± SEM. * *p* < 0.05; ** *p* < 0.01; *** *p* < 0.001, analyzed by one‐way ANOVA with Tukey's multiple comparison test (L).

Given robust NLRP3 induction in psoriatic groups, we next sought to elucidate upstream regulation of its expression. An AU located in the 3′UTR of *Nlrp3* mRNA contributes to its post‐transcriptional regulation by recruiting specific RBPs (Figure S19F). We therefore screened RBP gene sets annotated for 3'UTR binding of *Nlrp3* mRNA. From 126 candidates, 32 were AU binding and 9 linked to “3′UTR‐mediated mRNA destabilization”. Restricting to candidates with mean expression ≥ 10 in sham group yielded six RBPs (*Hnrnpd*, *Khsrp*, *Zc3h12a*, *Zfp36*, *Zfp36l1*, *Zfp36l2*) (Figure 5J). Among these, *Hnrnpd* (hereafter referred to as *Auf1*) and *Khsrp* mRNA were down‐regulated in IMQ and rIL‐23 groups but restored by RIMO, with *Auf1* showing the most consistent reversal (Figure 5K). Collectively, the RNA‐seq data identify AUF1 as a putative post‐transcriptional regulator of *Nlrp3* mRNA, thereby linking RBP down‐regulation to NLRP3 inflammasome activation. Consistently, in IMQ‐inflamed dorsal skin, CB1R was elevated, AUF1 was reduced, and NLRP3 increased compared with normal controls—all of which were reversed by RIMO (Figure 5L). Consistent with elevated NLRP3, cleaved caspase‐1 and mature IL‐1β—readouts of active NLRP3 inflammasome—were increased in IMQ‐inflamed skin and reduced by RIMO (Figure 5L). Human RNA‐seq cohorts revealed lower *Auf1* mRNA in psoriatic lesional skin compared with non‐lesional skin (Figure S20A). In murine macrophages, *Auf1* mRNA was decreased by the EC anandamide (AEA) and this effect was blocked by RIMO (Figure S20B,C). In contrast, *Nlrp3* mRNA levels were higher in psoriatic lesional skin compared with non‐lesional skin (Figure S20D), and LPS‐induced *Nlrp3* mRNA expression was suppressed by *Cnr1* deficiency and by RIMO (Figure S20E,F). In agreement with previous reports establishing NLRP3 dependence of psoriasis‐like skin dermatitis [58, 59, 60, 61, 62, 63], we found that *Nlrp3* deficiency markedly attenuated IMQ‐induced epidermal hyperplasia and skin inflammation; importantly, anti‐psoriatic effects of RIMO were abolished in *Nlrp3* ^−/−^ mice (Figure S21A,B). Together, these data identify the AUF1–NLRP3 axis as a major downstream pathway associated with CB1R‐dependent regulation of psoriatic skin inflammation.

### 2.6 AUF1 Represses *Nlrp3* mRNA via 3′UTR Binding to Attenuate Psoriasis‐Like Phenotypes

To test whether AUF1 directly interacts with *Nlrp3* mRNA, we carried out ribonucleoprotein immunoprecipitation (RIP) analysis in BMDMs and found significant enrichment of *Nlrp3* mRNA in AUF1‐immunoprecipitated complexes compared with IgG control (Figure 6A). To identify the specific binding region, we synthesized biotin‐labeled RNA probes corresponding to the *Nlrp3* 3′UTR and its AU. Using streptavidin affinity pull‐down assays, AUF1 was found to selectively bind the *Nlrp3* 3′UTR and its AU, whereas no interaction was detected with the control 3′UTR derived from *Gapdh* mRNA (Figure 6B). To assess functional relevance, we constructed reporters in which the *Nlrp3* 3′UTR AU was cloned downstream of the *EGFP* stop codon (pEGFP–*Nlrp3* 3′UTR AU) (Figure 6C). In HEK293T cells, *Auf1* silencing (siAuf1) selectively increased EGFP expression from the pEGFP–*Nlrp3* 3′UTR AU reporter but not from the control pEGFP–C1 (Figure 6D), indicating AUF1‐dependent repression mediated through the *Nlrp3* 3′UTR AU region. Furthermore, mRNA half‐life analysis in BMDMs showed that *Nlrp3* mRNA stability increased upon *Auf1* silencing (Figure 6E). Consistently, AUF1 knockdown enhanced LPS‐induced NLRP3 protein expression and *Nlrp3* mRNA levels (Figure 6F,G). Together, these results identify AUF1 as a destabilizing regulator of *Nlrp3* mRNA.

**FIGURE 6:**
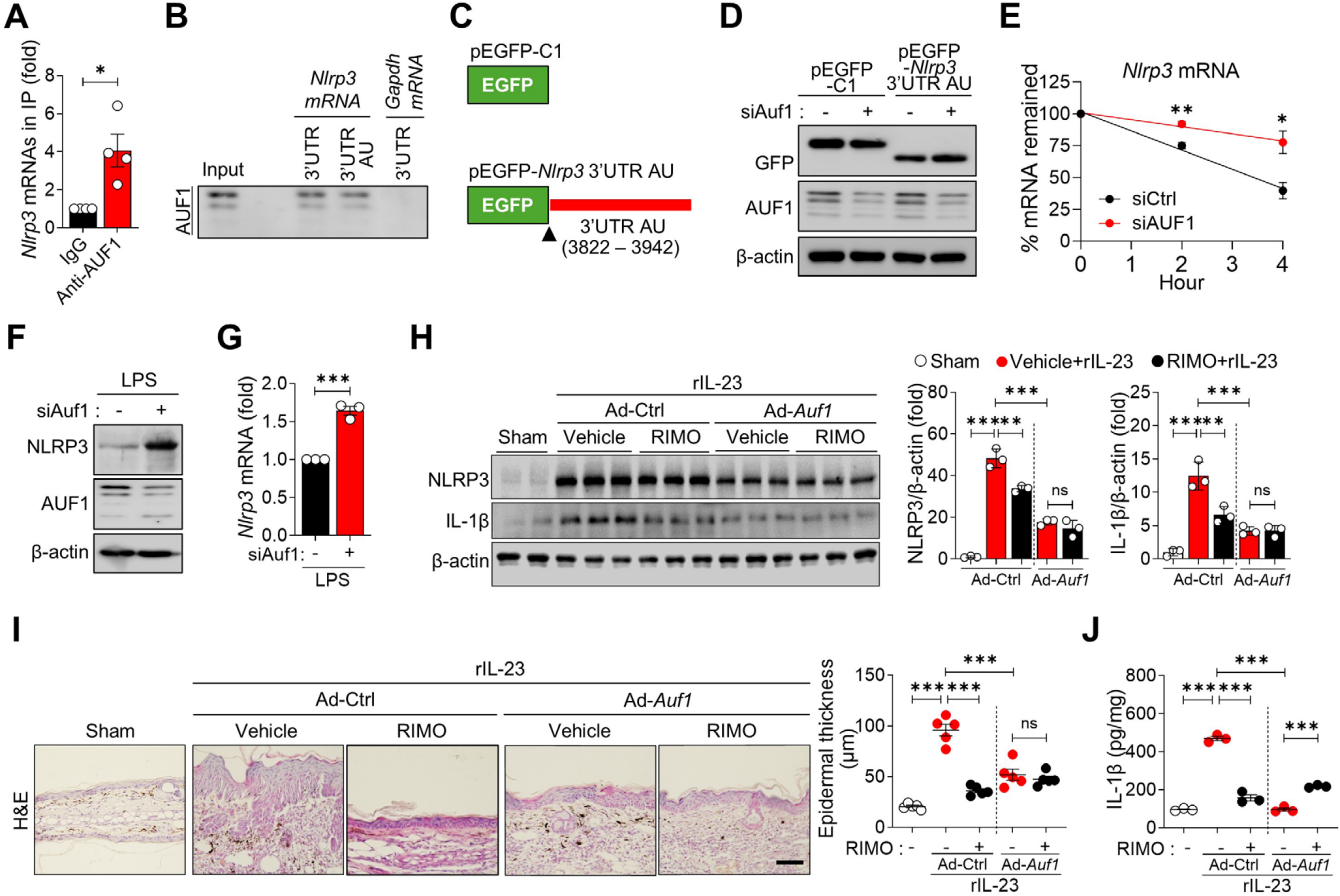
AUF1 binds the *Nlrp3* 3′ UTR and reduces *Nlrp3* expression. (A) RNA immunoprecipitation (RIP) in BMDMs using anti‐AUF1 or control IgG, followed by qPCR for *Nlrp3*; values normalized to *Gapdh* (*n*  =  4). (B) Biotin pull‐down of AUF1 with biotinylated RNA fragments corresponding to the full 3′ UTR and the AU of *Nlrp3* mRNA in RAW264.7 cells; biotinylated *Gapdh* 3′UTR served as a negative control. (C) Schematic of EGFP reporter plasmids: Parent vector (pEGFP–C1, top) and pEGFP–*Nlrp3* 3′UTR AU (bottom); black triangle indicates the stop codon. (D) Western blots of EGFP and AUF1 in HEK293T cells. Cells were transfected with siRNA (siCtrl or siAuf1) for 24 h, then with the indicated EGFP reporters; protein levels were assessed 24 h later. β‐actin, loading control. (E) The RNA decay curves of EU‐labeled *Nlrp3* mRNAs in BMDMs transfected with siCtrl or siAuf1. Time ‘0’ represents the input fully modified EU‐mRNA (*n* = 3). (F) Western blots of NLRP3 and AUF1 in RAW264.7 cells transfected with siCtrl or siAuf1 and stimulated with LPS (1 µg mL^−1^); β‐actin, loading control. (G) *Nlrp3* mRNA levels in RAW264.7 cells treated as in (F); qPCR normalized to *Gapdh* (*n*  =  3). (H) Western blots of NLRP3 and IL‐1β in ear skin on day 17; β‐actin, loading control. Densitometric quantification normalized to β‐actin is shown at right (*n*  =  3 mice per group). (I) Representative H&E images of ear sections on day 17 with quantification of epidermal thickness at right (*n*  =  5 mice per group). Scale bars, 100 µm. (J) ELISA quantification of IL‐1β in ear‐skin homogenates on day 17 (*n*  =  3 mice per group). Data are presented as mean ± SEM. *P < 0.05; **P < 0.01; ***P < 0.001 analyzed by two‐tailed student's *t* test (A, G) or two‐way ANOVA with Tukey's multiple comparison test (H–J), ns, not significant.

We next tested whether AUF1 suppresses NLRP3 expression in vivo and thereby mitigates psoriasis‐like phenotypes. Because AUF1 expression was reduced in IMQ‐ and rIL‐23‐inflamed skin, we used an adenoviral vector expressing AUF1 (Ad‐*Auf1*) to restore AUF1 in the rIL‐23‐inflamed mice. Eight days after adenoviral infection, mice receiving Ad‐*Auf1* showed markedly elevated AUF1 expression in the skin, without significant changes in body weight or evidence of toxicity in major organs compared with control adenovirus‐infected (Ad‐Ctrl) mice (Figure S22A–D). Accordingly, mice received rIL‐23 (intradermal) and RIMO (intraperitoneal) daily for 8 d starting on day 8 after infection (Figure S22A). In Ad‐Ctrl‐infected mice, RIMO suppressed rIL‐23‐induced NLRP3 and IL‐1β expression in skin (Figure 6H), consistent with the results shown in Figure 5L. In contrast, this rIL‐23‐induced NLRP3 and IL‐1β expression was markedly reduced and RIMO failed to exert additional inhibitory effects in Ad‐*Auf1*‐infected mice (Figure 6H). Likewise, Ad‐*Auf1*‐infected mice displayed significantly reduced epidermal hyperplasia and IL‐1β production compared with Ad‐Ctrl mice, while the therapeutic effects of RIMO were largely abolished (Figure 6I,J). Thus, these findings demonstrate that AUF1 represses NLRP3 expression by directly binding to its 3′UTR AU, thereby limiting inflammasome activation and psoriatic skin inflammation. Moreover, the loss of RIMO efficacy in AUF1‐overexpressing mice indicates that CB1R‐blockade benefits are, at least in part, AUF1‐dependent.

### 2.7 CB1R Regulates Psoriatic Skin Inflammation Through the AUF1–NLRP3 Axis In Vivo

Finally, to determine whether CB1R contributes to psoriasis pathogenesis via the AUF1–NLRP3 axis in vivo, we infected WT and *Nlrp3* ^−/−^ mice with either Ad‐Ctrl or Ad‐*Auf1*. Eight days after infection, mice were treated with intradermal rIL‐23 and intraperitoneal RIMO daily for eight consecutive days (Figure 7A). Consistent with the results shown in Figure 6H–J, compared with Ad‐Ctrl‐infected WT mice, Ad‐*Auf1*‐infected WT mice exhibited markedly attenuated rIL‐23‐induced epidermal hyperplasia, pro‐inflammatory cytokine expression, and macrophage infiltration (Figure 7B,C; Figure S23A–D). Notably, the protective effects of RIMO on rIL‐23‐induced clinical symptoms and skin inflammation were largely abolished in Ad‐*Auf1*‐infected WT mice (Figure 7B,C; Figure S23A–D). In addition, compared with WT mice, *Nlrp3* ^−/−^ mice displayed substantially reduced rIL‐23‐induced disease phenotypes (Figure 7B,C; Figure S23A–D), and neither AUF1 overexpression nor RIMO treatment further alleviated rIL‐23‐induced disease phenotypes in *Nlrp3* ^−/−^ mice (Figure 7B,C; Figure S23A–D). These findings identify AUF1 and NLRP3 as major downstream mediators of CB1R signaling and support the CB1R–AUF1–NLRP3–IL‐1β axis as a key pathway regulating psoriasis‐like skin inflammation in vivo.

**FIGURE 7:**
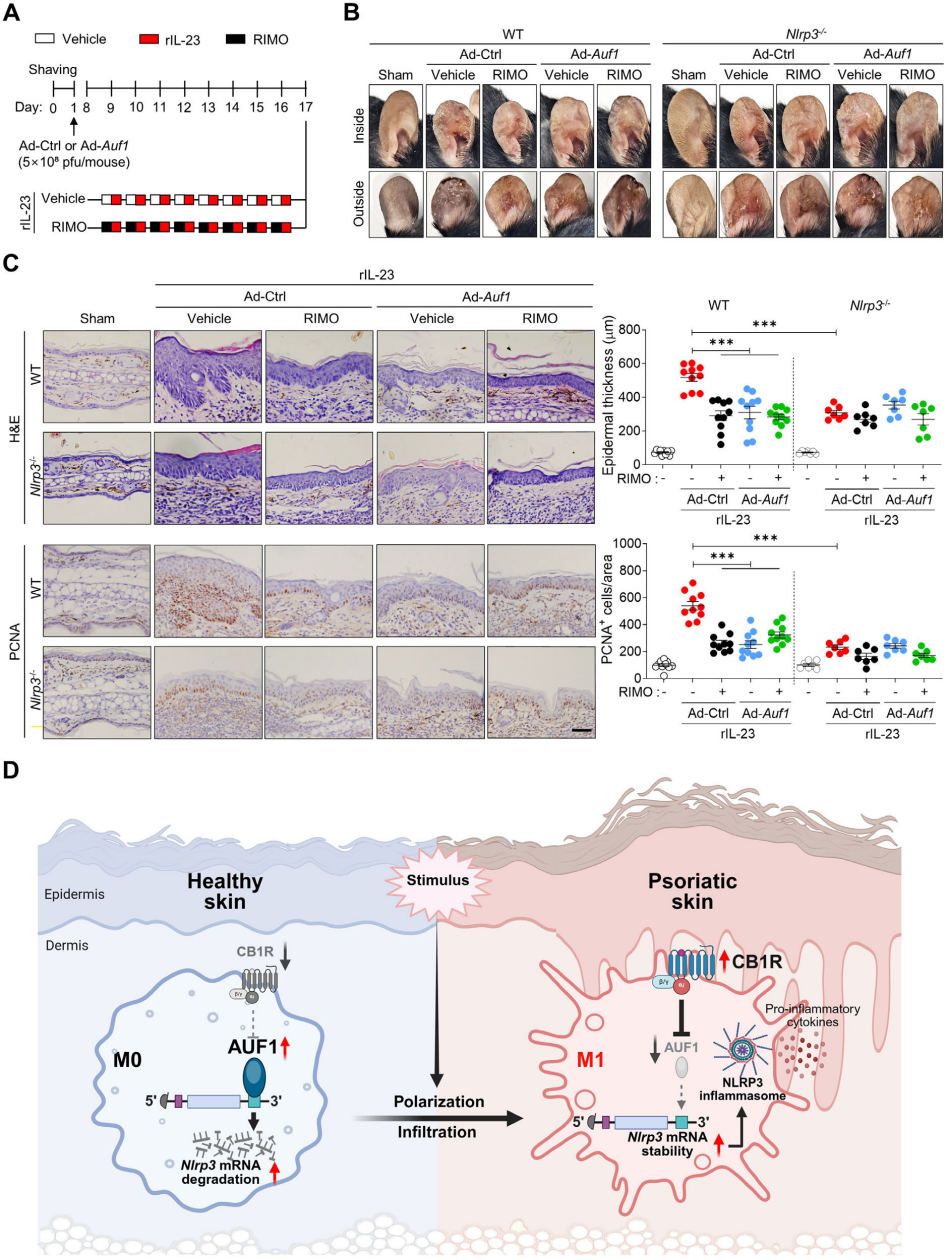
The CB1R–AUF1–NLRP3 axis mediates the anti‐psoriatic effects of RIMO in rIL‐23‐inflamed mice. (A) Experimental scheme. Ad‐Ctrl or Ad‐*Auf1* was injected intradermally into the ear on day 1. From days 9–16, rIL‐23 (500 ng) was administered once daily to induce psoriasiform dermatitis; RIMO (10 mg kg^−1^) or vehicle was given intraperitoneally 30 min before each rIL‐23 injection using a preventive treatment protocol. (B) Representative gross images of ears on day 17. (C) Representative H&E images (top) and PCNA immunohistochemistry (bottom) of ear sections on day 17. Epidermal thickness and the percentage of PCNA‐positive epidermal cells are shown at right (*n*  =  7–10 mice per group). Scale bars, 100 µm. (D) Schematic model of the CB1R–AUF1–NLRP3 axis regulating macrophage M1 polarization in healthy versus psoriatic skin: CB1R activation suppresses AUF1, stabilizes *Nlrp3* mRNA, and promotes IL‐1β production, whereas CB1R blockade restores AUF1, destabilizes *Nlrp3*, and limits inflammation. Data are presented as mean ± SEM. *** *p* < 0.001, analyzed by two‐way ANOVA with Tukey's multiple comparison test (C).

## 3 Discussion

Here, we identify macrophage CB1R as a critical upstream regulator of psoriatic skin inflammation and define the AUF1–NLRP3 axis as a previously unrecognized mechanism linking endocannabinoid signaling to post‐transcriptional control of innate immune activation (Figure 7D). Pharmacological inhibition or genetic deletion of CB1R attenuated psoriasis‐like inflammation in both IMQ‐ and rIL‐23‐induced models, restored epidermal barrier integrity, and suppressed pro‐inflammatory macrophage responses. Mechanistically, CB1R activation reduced AUF1 expression, thereby stabilizing *Nlrp3* mRNA and promoting NLRP3 inflammasome activation. Conversely, CB1R inhibition restored AUF1, enhanced *Nlrp3* mRNA decay, and reduced inflammasome‐dependent IL‐1β production. The functional importance of this pathway was further supported by AUF1 overexpression and *Nlrp3* deficiency. Together, these findings establish macrophage CB1R as a disease‐promoting regulator of psoriasis‐like inflammation and reveal post‐transcriptional control of *Nlrp3* mRNA as a key downstream mechanism.

CB1R has been extensively studied in metabolic tissues, where excessive CB1R signaling contributes to metabolic dysfunction and chronic inflammation. Pharmacological blockade or genetic deletion of CB1R improves insulin sensitivity, reduces adipose inflammation, and suppresses pro‐inflammatory cytokine production in models of obesity and T2D [17, 19, 23, 24, 39, 40], with related protective effects reported in the liver, pancreatic islets, kidney, and myocardium [19, 20, 25, 26, 41, 42, 43, 64, 65, 66, 67, 68]. Our findings extend this disease‐promoting function of CB1R to macrophage‐driven cutaneous inflammation. However, the role of CB1R in skin inflammation is likely to be cell‐type dependent. Liu et al. reported that sensory neuron‐specific *Cnr1* deletion exacerbated IMQ‐induced psoriasiform inflammation and pruritus, supporting a protective role for neuronal CB1R in neuroimmune regulation [29]. Rather than being mutually exclusive, these findings indicate that CB1R can exert opposing functions across cellular compartments: sensory neuronal CB1R may restrain neurogenic inflammation, whereas our data demonstrate that macrophage CB1R promotes inflammatory activation. Systemic CB1R inhibition necessarily integrates these cell‐specific effects. In our models, RIMO consistently attenuated IMQ‐ and rIL‐23‐induced inflammation, including when administered after disease establishment, and similar protection was observed with AM251 and several peripherally restricted CB1R antagonists. Moreover, global and myeloid lineage‐specific *Cnr1* deletion produced broadly comparable protection, while WT, but not *Cnr1* ^−/−^, BMDM reconstitution substantially restored disease in macrophage‐depleted mice. Thus, under the conditions examined, the aggregate effect of CB1R blockade is anti‐inflammatory, consistent with a substantial disease‐promoting contribution from peripheral myeloid/macrophage CB1R. These observations underscore the importance of cellular context in determining the inflammatory consequences of CB1R signaling.

A central mechanistic finding of our study is the identification of AUF1 as a post‐transcriptional regulator of NLRP3 inflammasome activation. RBPs are increasingly recognized as critical determinants of macrophage inflammatory responses by controlling the stability and translation of transcripts encoding cytokines and other inflammatory mediators [30, 31, 32]. AUF1 promotes the degradation of several inflammatory mRNAs, including those encoding TNF‐α and IL‐6, and AUF1 deficiency causes systemic hyperinflammation [33, 34, 35, 36, 37, 38]. Our findings extend this paradigm by identifying *Nlrp3* mRNA as a direct AUF1 target. AUF1 bound the *Nlrp3* 3′UTR and accelerated *Nlrp3* mRNA decay, whereas loss of AUF1 enhanced NLRP3 expression and inflammasome activation. Moreover, the absence of additional protection by AUF1 overexpression in *Nlrp3* ^−/−^ mice places NLRP3 as a major functional downstream effector of AUF1 in this pathway. Because NLRP3‐dependent IL‐1β production amplifies the IL‐23/IL‐17 inflammatory circuit in psoriasis [58, 59, 60, 61, 62, 63], the CB1R–AUF1 pathway provides a mechanism through which EC signaling can regulate a key inflammatory amplification loop at the level of mRNA stability. Nevertheless, AUF1 is a pleiotropic RBP with numerous inflammatory targets, and additional AUF1‐regulated transcripts may contribute to the observed phenotype. Transcriptome‐wide identification of AUF1‐bound RNAs will therefore be important for defining the broader post‐transcriptional network controlled by CB1R signaling. In addition, although our findings establish AUF1 as a functional mediator linking CB1R to *Nlrp3* mRNA destabilization, the receptor‐proximal mechanisms through which CB1R signaling suppresses AUF1 remain incompletely defined and warrant further investigation.

These findings may have particular relevance to the well‐established association between psoriasis and metabolic disease. Psoriasis frequently coexists with obesity, insulin resistance, and T2D [4, 5, 6, 8, 9, 10], conditions characterized by chronic low‐grade inflammation and dysregulated EC signaling. Excessive peripheral CB1R activity contributes to metabolic dysfunction and macrophage‐driven inflammation in obesity and T2D, whereas the present study demonstrates that macrophage CB1R also promotes cutaneous inflammation through post‐transcriptional regulation of the AUF1–NLRP3 axis. These observations raise the possibility that macrophage CB1R represents a shared regulatory node connecting metabolic and cutaneous inflammation. In this framework, excessive EC–CB1R signaling could enhance NLRP3 inflammasome activity by suppressing AUF1‐dependent *Nlrp3* mRNA decay, thereby sustaining macrophage inflammatory responses across metabolically and immunologically distinct tissues. Although direct demonstration of this mechanism in patients with concurrent psoriasis and metabolic disease will require further investigation, our findings provide a mechanistic framework for understanding how a metabolic signaling pathway can converge on innate immune regulation in psoriasis.

From a translational perspective, our findings support further investigation of peripherally restricted CB1R antagonism as a therapeutic strategy for psoriasis. RIMO was used primarily as a pharmacological tool to define the CB1R–AUF1–NLRP3 pathway, but its brain penetration and neuropsychiatric adverse effects limit its clinical utility. Importantly, several peripherally restricted CB1R antagonists reproduced its anti‐psoriatic effects in our models, indicating that brain CB1R blockade is not required for therapeutic efficacy. This conclusion is further supported by the protective phenotype of myeloid lineage‐specific *Cnr1* deletion and by macrophage‐reconstitution experiments demonstrating a macrophage‐intrinsic contribution of CB1R to disease. Peripherally restricted antagonists therefore offer a potential means to retain CB1R blockade in peripheral tissues, including immune compartments, while limiting brain exposure and the CNS liabilities associated with first‐generation antagonists. Such an approach may be particularly attractive for psoriasis accompanied by metabolic comorbidities, because peripheral CB1R inhibition could potentially modulate inflammatory and metabolic pathways that contribute to both conditions. Nevertheless, long‐term efficacy, pharmacokinetics, CNS exposure, and neuropsychiatric safety will require rigorous evaluation before the therapeutic potential of this approach can be established.

Several limitations should be considered. First, our in vitro mechanistic studies relied primarily on BMDMs, which provide a genetically tractable system but do not fully recapitulate the transcriptional, epigenetic, and functional heterogeneity of macrophage populations in human or mouse skin. Although the in vitro findings are supported by increased CB1R expression in macrophages within inflamed skin, myeloid lineage‐specific *Cnr1* deletion, and macrophage‐reconstitution experiments, future lineage‐tracing and subset‐specific approaches will be required to distinguish the contributions of infiltrating monocyte‐derived and tissue‐resident macrophages. Second, the opposing function of sensory neuronal CB1R reported previously was not directly examined in our study. Studies combining sensory neuron‐specific *Cnr1* deletion with pharmacological CB1R inhibition will be important for defining the relative contributions of neuronal and immune‐cell CB1R to the net therapeutic response. Third, although our human analyses support increased macrophage‐associated CB1R expression in psoriatic lesions, larger and clinically stratified cohorts will be required to determine how CB1R expression relates to disease severity, metabolic comorbidities, and therapeutic response. Finally, the upstream signaling mechanisms linking CB1R activation to AUF1 suppression remain to be fully resolved. Addressing these questions will further define the cellular and molecular contexts in which the CB1R–AUF1–NLRP3 axis contributes to human psoriasis.

## 4 Conclusion

In summary, our study identifies macrophage CB1R as a disease‐promoting regulator of psoriatic inflammation and reveals AUF1‐dependent destabilization of *Nlrp3* mRNA as a key post‐transcriptional mechanism linking CB1R signaling to inflammasome activation. These findings establish a previously unrecognized CB1R–AUF1–NLRP3 axis that connects EC signaling with macrophage innate immune responses in psoriasis. The efficacy of peripherally restricted CB1R antagonists further supports peripheral CB1R as a potential therapeutic target, particularly in the context of psoriasis associated with metabolic comorbidities. Together, our findings provide a mechanistic and translational framework for targeting macrophage CB1R in psoriasis.

## 5 Materials and Methods

Information on reagents, primers, antibodies, and other resources is summarized in Table S1.

### 5.1 Animals

All animal experiments were approved by the Institutional Animal Care and Use Committee (IACUC) of Ajou University (No. 2023‐0031) and complied with relevant regulations. *Nlrp3* ^−/−^ (The Jackson Laboratory, Bar Harbor, ME, USA; Jax 021302) mice on a C57BL/6J background were obtained from The Jackson Laboratory. All strains used in this study (WT, *Cnr1* ^flox/flox^, *CMV‐Cre*, *LysM‐Cre, Cnr1* ^−/−^, and M‐*Cnr1* ^−/−^) were maintained on a C57BL/6J background. Animals were housed under specific‐pathogen‐free conditions at Ajou University with ≤5 mice per cage, controlled temperature (24 ± 2°C) and humidity (50 ± 10%), and a 12‐h light/dark cycle, with ad libitum access to water and standard chow. For in vivo experiments, age‐ and sex‐matched mice were cohoused for ≥1 week and then randomized to experimental groups. All animal studies were performed with randomized group assignment and blinded outcome assessment, with sample sizes determined based on prior experiments to ensure reproducibility and statistical power. To study skin inflammation and psoriasis in vivo, we exclusively used female mice because they exhibit lower aggression and develop more consistent and reproducible psoriasis‐like phenotypes following IMQ or rIL‐23 treatment compared with males, thereby reducing experimental variability.

### 5.2 Generation of *Cnr1* Mutant Mice and Genotyping

*Cnr1* ^flox/flo^ *^x^* mice were generated as previously described [69]. In this study, *Cnr1* ^−/−^ mice were produced by crossing *Cnr1* ^flox/flox^ with *CMV*‐*Cre* mice; M‐*Cnr1* ^−/−^ mice were obtained by crossing *Cnr1* ^flox/flox^ with *LysM*‐*Cre* mice. Genotypes were verified by standard PCR using genomic DNA from BMDMs. Primer sets were as follows: LOX1 (5′‐CTGGACAGCTCATCCCTTGGTTCTATAC‐3′), SDL2 (5′‐TGAGTGGCAAGAAAAG TCAGAGTG‐3′), and NDL2 (5′‐CAAGAGACTGCAGCAGGCCAAATCTAG‐3′). PCR amplification with the LOX1/NDL2 primer pair detected the recombined *Cnr1*Δ allele (397 bp), whereas the LOX1/SDL2 primer pair distinguished wild‐type (427 bp) and floxed (487 bp) alleles.

### 5.3 Human Samples

Human validation was performed using lesional and non‐lesional skin biopsies from patients with psoriasis. Human skin samples were collected at Seoul St. Mary's Hospital, The Catholic University of Korea, from donors who provided written informed consent, under protocols approved by the Institutional Review Board of the Catholic University of Korea (IRB no. KC22SISI0408). Donor characteristics are summarized in Table S2.

### 5.4 Cell Lines and Primary Cell Cultures

RAW264.7 (mouse macrophage‐like; ATCC, Manassas, VA, USA; TIB‐71) and HEK293T (human embryonic kidney; ATCC, CRL‐3216) cells were cultured in DMEM (Capricorn Scientific, Ebsdorfergrund, Germany; DMEM‐HPA) supplemented with 10% FBS (Thermo Scientific, Waltham, MA, USA; A5670801) and 1% penicillin/streptomycin (HyClone, Logan, UT, USA; PS‐B) at 37°C in 5% CO_2_. For primary BMDMs, bone marrow from 6–8‐week‐old male C57BL/6 mice was flushed with RPMI‐1640 (Capricorn Scientific, RPMI‐A), red blood cells (RBCs) were lysed with RBC lysis buffer (eBioscience, San Diego, CA, USA; 00‐4333‐57), and cells were cultured for 6 d in RPMI‐1640 supplemented with 10% FBS, 1% penicillin/streptomycin, and 20 ng mL^−1^ M‐CSF (Sino Biological, Beijing, China; 51112‐MNAH), with medium changes every 3 d. For primary BMDCs, cells were cultured for 6 d in RPMI‐1640 supplemented with 10% FBS, 1% penicillin/streptomycin, and 40 ng mL^−1^ GM‐CSF (Abcam, Cambridge, UK; ab9742). Primary peritoneal macrophages were harvested by peritoneal lavage from 6–8‐week‐old male C57BL/6 mice and cultured in RPMI‐1640 with 10% FBS and 1% penicillin/streptomycin.

### 5.5 Generation of CB1R‐FAM

Anti‐CB1R antibody (Thermo Scientific, PA1‐743) was diluted to 0.02 mg mL^−1^ in PBS‐bicarbonate (100 mm, pH 8.4) and reacted with a 10‐fold molar excess of 5‐carboxyfluorescein‐C2TCO‐NHS linker for 3 h at room temperature. The reaction mixture was buffer‐exchanged and concentrated using 30‐kDa Amicon filters (EMD Millipore, Burlington, MA, USA; UFC5030) to remove free dye and salts, then adjusted to 0.2 mg mL^−1^. Degree of labeling (DOL) was determined by NanoDrop One (A280 and A490; dye and IgG extinction coefficients with CF280 applied; Thermo Scientific), yielding a DOL of 1.31. Probe specificity was validated by flow cytometry and confocal imaging in WT versus *Cnr1* ^−/−^ BMDMs.

### 5.6 Western Blotting Analysis

Skin tissues or cells were lysed in ice‐cold RIPA buffer (50 mm Tris‐HCl pH 7.4, 150 mm NaCl, 1% NP‐40, 0.1% SDS, and 1 mm EDTA; Invitrogen, Waltham, MA, USA; 78510) with protease inhibitors (Sigma‐Aldrich, QTPPI1015) and phosphatase inhibitors (Sigma‐Aldrich, P5726). Lysates were cleared at 12 000 × *g* for 10 min at 4°C, quantified by Bradford assay (Bio‐Rad, Hercules, CA, USA; BR500‐0002), resolved by SDS‐PAGE, and transferred to PVDF (Bio‐Rad, BR1620177). Membranes were probed with primary antibodies against CB1R (Cell Signaling, Danvers, MA, USA; 93815S, 1:500), AUF1 (EMD Millipore, 07–260, 1:1000), NLRP3 (Adipogen, Liestal, Switzerland; AG‐20B0014‐C100, 1:1000), Caspase‐1 (Cell Signaling, 89332S, 1:1000), IL‐1β (Novus Biologicals, Centennial, CO, USA, NB600‐633, 1:1000), Claudin‐1 (Abcam, ab15098, 1:2000), S100A9 (Novus Biologicals, NB110‐89726, 1:2000), GFP (Santa Cruz, Dallas, TX, USA; SC‐9996, 1:1000), and β‐actin (GeneTex, Irvine, CA, USA; GTX629630, 1:10000). HRP‐conjugated secondary antibodies and Clarity ECL (Glpbio, Montclair, CA, USA; GK10008) were used; signals were captured on a ChemiDoc (Bio‐Rad).

### 5.7 Cell Isolation from Skin

Skin tissues were rinsed in cold Ca^2+^/Mg^2+^‐free HBSS (Gibco, Waltham, MA, USA; 14025–092), minced, and digested in DMEM containing 200 U mL^−1^ collagenase D (Sigma‐Aldrich) and 0.01 mg mL^−1^ Liberase TL (Sigma‐Aldrich, 5401020001) for 1.5 h at 37°C with gentle agitation. Cell suspensions were passed through a 40‐µm pore cell strainer (SPL Life Sciences, Pocheon, Korea; 93040), subjected to RBC lysis, washed, and resuspended in staining buffer (PBS, 5 mM EDTA, 2% BSA, and 2% FBS) for flow cytometry.

### 5.8 RNA Immunoprecipitation

Ribonucleoprotein complexes were immunoprecipitated from cell lysates using Protein A beads (Invitrogen, 15918‐014) pre‐bound with anti‐AUF1 IgG (EMD Millipore, 07‐260) or control rabbit IgG (Sigma‐Aldrich, 12‐370). After DNase I (Thermo Scientific, EN0521) and proteinase K (Thermo Scientific, EO0491) treatment, RNA was purified, reverse‐transcribed, and analyzed by qPCR with gene‐specific primers (^ΔΔ^Ct; *Gapdh* mRNA as control). Primer sequences are listed in Table S3.

### 5.9 IMQ‐ and rIL‐23‐Induced Psoriasis Models

Seven‐week‐old female C57BL/6 mice were acclimated for one week before experiments. For the IMQ‐induced psoriasis model, 5% IMQ (Aldara; 3 M Pharmaceuticals, Maplewood, MN, USA) was applied topically to the shaved dorsal skin at a dose of 62.5 mg cm^−^^2^ once daily for four consecutive days. RIMO (10 mg kg^−1^, intraperitoneally) or other CB1R antagonists—AM251 (Sigma‐Aldrich, A6226; 10 mg kg^−1^), AM6545 (Sigma‐Aldrich, A1987, 10 mg kg^−1^), JD5037 (MCE, Monmouth Junction, NJ, USA; HY‐18697; 10 mg kg^−1^), TM38837 (MCE; HY‐112340; 10 mg kg^−1^) or BPRCB1184 (MCE; HY‐12790; 10 mg kg^−1^)—were administered 30 min prior to each daily IMQ application. In the delayed‐treatment protocol, mice received topical IMQ for four days followed by two intraperitoneal injections of RIMO (10 mg kg^−1^) beginning on day 3. For the rIL‐23‐induced psoriasis model, 500 ng of mouse rIL‐23 (eBioscience, 14‐8231‐63) dissolved in 20 µL of PBS containing 1% BSA was injected intradermally into the ear skin once daily for eight consecutive days. Ad‐*Auf1* (Vector Biolabs, Malvern, PA, USA; ADV‐211371; 5×10^8^ pfu per mouse) or Ad‐Ctrl (Vector Biolabs; 1300; 5×10^8^ pfu per mouse) was injected intradermally 8 d before rIL‐23 administration, and RIMO (10 mg kg^−1^, intraperitoneally) was given 30 min prior to each daily rIL‐23 injection. The severity of psoriasis‐like phenotypes was assessed daily using a PASI‐like scoring system evaluating erythema, scaling, and thickness on a 0–4 scale (none = 0, mild = 1, moderate = 2, severe = 3, very severe = 4), yielding a total score ranging from 0 to 12. At the end of the experiment, mice were euthanized, and full‐thickness skin samples were collected for histological examination, immunostaining, flow cytometry, and gene expression analyses.

### 5.10 In Vivo Skin Penetration Assay

To assess skin barrier function, 4‐kDa FITC‐dextran (5 mg cm^−^^2^; Sigma‐Aldrich, FD4) was applied on gauze to shaved dorsal skin for 6 h under an occlusive dressing (Tegaderm, 3 M Pharmaceuticals) on day 5 after IMQ ± RIMO treatment. Serum FITC fluorescence (Ex 485 nm/Em 528 nm) was measured using a multi‐well spectrophotometer (Cytation 3, BioTek Instruments Inc., Winooski, VT, USA), and FITC‐dextran concentrations were determined using a standard curve generated by serial dilution of FITC‐dextran in mouse plasma.

### 5.11 Histology, Immunostaining, Immunohistochemistry and Image Processing/Analysis

Skin and brain tissues were fixed in 4% paraformaldehyde (BIOSESANG, Seongnam, Korea; PC2031), paraffin‐embedded, and sectioned (4 µm). For histology, sections were stained with hematoxylin (EMS, Hatfield, PA, USA; 26602‐02) and eosin Y (EMS; 26051‐11) using standard procedures. For immunostaining and immunohistochemistry, sections were deparaffinized, rehydrated, antigen‐retrieved (10 mm sodium citrate, pH 6.0, 30 min at ≈95–100°C; Invitrogen, 00‐4956‐58), permeabilized with 0.25% Triton X‐100 (Sigma‐Aldrich, T9284), and blocked in normal goat serum blocking solution (Vector Laboratories, Newark, CA, USA; PK‐4001). Primary antibodies (4°C overnight) included CB1R (Cell Signaling, 93815S, 1:200), CD4 (R&D Systems, Minneapolis, MN, USA; MAB554, 1:200), CD68 (R&D Systems, MAB101141‐100, 1:200), CD86 (APC; Invitrogen, 17‐0862‐81, 1:200), CD206 (APC; Invitrogen, 17‐2061‐82, 1:200), F4/80 (Novus Biologicals, NB600‐404, 1:500), IL‐1β (Novus Biologicals; NB600‐633, 1:500), IL‐17A (Novus Biologicals; NBP1‐72027, 1:200), IL‐23 (Novus Biologicals; NBP1‐76697, 1:500), involucrin (BioLegend, San Diego, CA, USA; 924401, 1:3000), Ki‐67 (Cell Signaling, 12202S, 1:200), loricrin (Abcam, ab85679, 1:3000), PCNA (Santa Cruz, SC‐56, 1:1500) and TNF‐α (Novus Biologicals; NBP1‐19532, 1:1500). For immunostaining, slides were incubated for 1 h at room temperature with Alexa Fluor 488 goat anti‐rat IgG (Invitrogen, A11006, 1:1000), Alexa Fluor 546 goat anti‐mouse IgG (Invitrogen, A11030, 1:1000), Alexa Fluor 546 goat anti‐rat IgG (Invitrogen, A11081, 1:1000) or Alexa Fluor 633 goat anti‐rabbit IgG (Invitrogen, A21070, 1:1000) followed by counterstaining with ProLong Gold Antifade Reagent with DAPI (Invitrogen, R37606, 1:2000). Images were acquired on a Leica DMi8 (Leica Microsystems, Wetzlar, Germany) or Leica Stellaris5 confocal microscopy equipped with a 40× and 63× objectives and analyzed in ImageJ (National Institutes of Health, Bethesda, MD, USA). For immunohistochemistry, biotinylated secondaries (Vector Laboratories, 1:200) and Vectastain Elite ABC with DAB (Vector Laboratories, SK4100) were used, followed by hematoxylin counterstain. Epidermal thickness was quantified by drawing ≥30 perpendicular line segments from stratum corneum to the dermo‐epidermal junction per section (ImageJ MRI skin tools). At least nine fields from ≥3 sections per mouse were analyzed for signal intensity.

### 5.12 In Vivo Macrophage Depletion

Macrophages were depleted using clodronate‐encapsulated liposomes (FormuMax Scientific Inc., Sunnyvale, CA, USA; F70101C‐NC). Mice received 200 µL (1.4 mg) clodronate liposomes or control liposomes intraperitoneally 1 day before IMQ (3 M Pharmaceuticals, Aldara) application, followed by 100 µL (0.7 mg) on day 3. Macrophage depletion was confirmed by immunohistochemistry (CD68) and by flow cytometry of peritoneal macrophages.

### 5.13 Macrophage Adoptive Transfer

BMDMs were isolated from WT and *Cnr1*−/− mice and differentiated for 7 d. Prior to adoptive transfer, BMDMs were labeled with CellTrace Far Red Cell Proliferation Dye (Thermo Fisher Scientific; C34564) according to the manufacturer's instructions. For macrophage depletion, mice received a single subcutaneous injection of clodronate liposomes (200 µL per mouse) into the dorsal skin 2 d before the first IMQ application. On the first day of IMQ treatment, Far Red‐labeled WT or *Cnr1*−/− BMDMs (4 × 10^6^ cells per mouse) were administered subcutaneously into the dorsal region 4 h before IMQ application. IMQ cream was subsequently applied to the shaved dorsal skin once daily for four consecutive days, as described above. At the end of the experiment, the presence of Far Red‐labeled BMDMs in the dorsal skin was assessed by Leica Stellaris5 confocal microscopy.

### 5.14 Plasmids, siRNA, and Transfection

An EGFP reporter (pEGFP–*Nlrp3* 3′UTR AU) was generated by cloning the AU of the *Nlrp3* 3′UTR (nt 3822–3942; 121 nt; NM_145827.4) downstream of EGFP in pEGFP‐C1 (BD Biosciences, 6084‐1). HEK293T or RAW264.7 cells were transfected with siRNAs (siCtrl and siAuf1) or plasmids using Lipofectamine reagent (Invitrogen, 11668019). siRNA sequences are provided in Table S4.

### 5.15 Adenovirus Infection

Ad‐*Auf1* (Ad‐CMV‐h‐HNRNPD) and Ad‐Ctrl were purchased from Vector Biolabs (Malvern, PA). For ear‐skin delivery, Ad‐*Auf1* (5×10^8^ pfu per mouse) or Ad‐Ctrl (5×10^8^ pfu per mouse) in PBS was injected intradermally 8 d prior to rIL‐23 treatment.

### 5.16 Flow Cytometry

Cells were blocked with anti‐CD16/32 (clone 2.4G2; BD Biosciences, San Jose, CA, USA) and stained with fluorophore‐conjugated antibodies on ice for 1 h. Acquisition was performed on a NovoCyte flow cytometer (Agilent Technologies, Santa Clara, CA, USA) and analyzed with NovoExpress software (Agilent Technologies). CD45^−^ and CD45^+^ fractions from skin were sorted on a BD FACSAria (BD Biosciences). For immune‐cell population analysis, single‐cell suspensions were stained with fluorochrome‐conjugated antibodies against CD3ε, CD8α, CD11b, CD11c, CD19, CD45, CD86, CD206, F4/80, and Ly6G. The following antibodies were used: anti‐mouse CD3ε (FITC; BioLegend, 53‐0031‐82), anti‐mouse CD8α (PE; Invitrogen, 12‐0081‐82), anti‐mouse CD11b (FITC; Invitrogen, 53‐0112‐82), anti‐mouse CD11c (PerCP; BioLegend, 117326), anti‐mouse CD19 (PE; Invitrogen,12‐0199‐42), anti‐mouse CD45 (APC‐Cy7; Invitrogen, A15395), anti‐mouse CD45 (PE; Invitrogen, 12‐0451‐83), anti‐mouse CD86 (APC; Invitrogen,17‐0862‐81), anti‐mouse CD206 (PE; Invitrogen, 14‐1706‐82), anti‐mouse CD206 (APC; Invitrogen), anti‐mouse F4/80 (PE‐Cy7; Invitrogen, 25‐4801‐82) and anti‐mouse Ly6G (PE‐Cy7; Invitrogen, 25‐5931‐82). All antibodies were diluted using 1:50 prior to staining.

### 5.17 RNA Extraction and qPCR

Total RNA was isolated with RNAiso Plus (Takara Bio, Shiga, Japan; 9109), and cDNA was synthesized using ReverTra Ace qPCR RT Kit (TOYOBO, Osaka, Japan; FSQ‐101). qPCR was performed with SensiFAST SYBR Hi‐ROX (Meridian Bioscience, Cincinnati, OH, USA; BIO‐90200) on a StepOnePlus Real‐Time PCR System (Applied Biosystems, Waltham, MA, USA). Relative expression was calculated by the ^ΔΔ^Ct method normalized to *Gapdh* mRNA. Primer sequences are listed in Table S3.

### 5.18 In Vitro Macrophage Polarization

BMDMs were treated with LPS (10 ng mL^−1^; Sigma‐Aldrich, L5293) or IMQ (1 µg mL^−1^; Invitrogen, R837) for 4 or 24 h with vehicle or RIMO (1 µm). RAW264.7 cells were stimulated with LPS (100 ng mL^−1^) ± RIMO (1 µm) for 4 h. For M2 polarization, BMDMs were treated with IL‐4 (20 ng mL^−1^; Novoprotein, CK15) for 24 h. Cells were analyzed by qPCR, flow cytometry, and/or immunostaining.

### 5.19 RNA Sequencing and Bioinformatics Analysis

RNA‐seq libraries were generated using the TruSeq mRNA Library Prep Kit (Illumina, Inc., USA) according to the manufacturer's instructions. Briefly, each 100 ng total RNA isolated immune cells from pooled dorsal skin (IMQ‐induced model) or pooled ear skin (IL23‐induced model) was used for library preparation. An oligo‐dT primer containing an Illumina‐compatible sequence at its 5’ end was hybridized to the RNA and reverse transcription was carried out. After degradation of the RNA template, second strand synthesis was initiated using a random primer containing an Illumina‐compatible linker sequence at its 5’ end. The resulting double‐stranded cDNA library was purified using AMPure XP magnetic beads (A63881, Beckman coulter, CA, USA) to remove reaction components. The library was PCR‐amplified to add the complete adapter sequences required for cluster generation, and the finished library was purified from PCR components. High‐throughput sequencing was performed as paired‐end 101 bp reads on an Illumina NovaSeq 6000 platform (Illumina, Inc., USA). Raw sequencing reads were first subjected to adapter and quality trimming using TrimGalore (v.0.6.6) with default parameters. The trimmed reads were then aligned to the mouse reference genome (GRCm38.p6.101) using STAR (v2.7.1a). Gene‐level counts and FPKM values were then obtained using RSEM (v.1.3.3). The count matrix was analyzed in R using DESeq2 (v.1.32.0) to calculate normalized FPKM value and to identify differentially expressed genes.

### 5.20 KEGG Pathway Analysis

KEGG enrichment was performed using DAVID (PMID: 35325185). Enriched KEGG terms were obtained for each of the four pairwise comparisons, and pathways detected in at least three of four comparisons were retained. Pathways were ranked by the mean ‐log10(*p* value) across the NC‐IMQ and NC‐IL23 comparisons. To prevent inflation of the ranking metric, *p* values ≤ 1×10^−4^ were capped at 1×10^−4^ prior to transformation.

### 5.21 Weighted Gene Co‐Expression Network Analysis (WGCNA) and Protein–Protein Interaction (PPI) Network Analysis

WGCNA was performed in R (v4.4.3) using the WGCNA package (v1.73) on genes with FPKM ≥ 50 in at least one sample. Samples were grouped as control (NC; pooled across IMQ and IL23 groups), psoriatic (IMQ and IL23), and RIMO‐treated, yielding a total of 12 samples. Modules were identified using the dynamic tree‐cutting algorithm, and modules of interest were selected based on their association with experimental groups. PPI networks for selected module genes were constructed with STRING database [PMID: 36370105]. Only experimentally supported or curated database interactions were extracted. After constructing the PPI networks, we intersected them with the WGCNA module genes and retained only the overlapping links. These integrated networks were then visualized and further analyzed using Cytoscape (v3.10.3).

### 5.22 Biotin Pull Down Assay

To synthesize biotinylated transcripts, DNA fragments corresponding to the *Nlrp3* (NM_145827.4) 3′UTR and its AU segment were amplified by PCR using forward primer containing T7 RNA polymerase binding sequence (5’‐CCAAGCTTCTAATACGACTCACTATAGGGAGA‐3’). After purification of the PCR products, biotinylate transcripts were synthesized using the EZ MEGA T7 Transcription Kit (enzynomics, EZ039S) and biotin‐CTP (Enzo Life Sciences, Farmingdale, NY, USA; ENZ‐42818). Cell lysates were incubated with the purified biotinylated transcripts for 30 min at room temperature, and the RNA–protein complexes were isolated using streptavidin‐coupled Dynabeads (Invitrogen, 11206D). Bound proteins were analyzed by western blotting for AUF1. *Gapdh* 3′UTR served as a negative control.

### 5.23 Measurement of mRNA Stability Using Click‐iT Chemistry

mRNA stability was assessed using the Click‐iT Nascent RNA Capture Kit (Invitrogen, C10365). BMDMs transfected with siRNA (siCtrl or siAUF1) were labeled with 200 µm ethynyl uridine (EU) at 37°C overnight. The medium was then replaced with EU‐free medium, and cells were harvested at 0, 2, and 4 h. Total RNA was isolated, and 500 ng of total RNA was subjected to the Click‐iT biotinylation reaction according to the manufacturer's instructions. Biotinylated RNA was captured using streptavidin magnetic beads. The captured RNA was reverse transcribed on the beads, and relative mRNA levels were quantified by qPCR. Transcript levels were normalized to *Gapdh* and expressed relative to the 0‐hour time point for each group using the ΔΔCt method. Primer sequences are listed in Table S3.

### 5.24 Elisa

TNF‐α, IL‐1β, IL‐17A, and IL‐23p19 proteins in skin homogenates was quantified using commercial ELISA kits (Thermo Scientific, 88‐7013‐76) according to the manufacturer's instructions.

### 5.25 Statistical Analysis

Data are expressed as the mean ± standard error of the mean (SEM). Statistical analyses were performed using GraphPad Prism (version 10.0.2; GraphPad Software, San Diego, CA, USA). Comparisons between two groups were conducted using an unpaired, two‐tailed Student's *t*‐test or Welch's corrected *t‐*test, as appropriate. For comparisons among more than two groups, one‐way followed by Tukey's multiple comparison test or two‐way ANOVA followed by Tukey's multiple comparison or Bonferroni's *post hoc* test was applied, as appropriate. Statistical significance was defined as *p* < 0.05 (* *p* < 0.05, ** *p* < 0.01, *** *p* < 0.001, ****p<0.0001). The number of biological replicates (n) is indicated in each figure legend. For in vitro experiments, each result represents at least three independent biological replicates, each performed in technical triplicate. All figure legends specify the statistical test used and the sample size for each group.

## Author Contributions

W.K., E.K.L., and J.W.K. conceived, designed, and supervised this study. H.C., S.H., and J.K. performed most experiments and analyzed data. J.H.H., J.L., H.R.Y., S.L., S.H.H., I.Y., S.A., and H.K. contributed to animal experiments. E.K., Y.H., and E.K. synthesized CB1R antagonists. M.L. contributed to the development of CB1R‐FAM. H.J.P. assisted with the analysis of RNA‐seq data. C.H.B. and J.H.L. contributed to human tissue analysis. J.M.E. provided *Cnr1*‐floxed mice and contributed to animal experiments. W.K., E.K.L., J.W.K., H.C., S.H., and J.K. wrote the manuscript. W.K., E.K.L., J.W.K., H.C., S.H., J.K., J.H.H., E.K., H.J.P., C.H.B., J.H.L., and J.M.E. revised and edited the paper.

## Funding

This study was supported by the National Research Foundation of Korea (NRF) grants (RS‐2023‐NR077133, RS‐2024‐00411474, RS‐2026‐25525938 and RS‐2026‐25482105 to W.K., RS‐2024‐00405790, RS‐2026‐25523717 and RS‐2026‐25473802 to E.K.L., and RS‐2026‐25558715 to H.C.), funded by the Ministry of Science and ICT, and by the Korea Drug Development Fund (RS‐2023‐00217308 to W.K.), funded by the Ministry of Science and ICT, the Ministry of Trade, Industry, and Energy, and the Ministry of Health and Welfare. This work was also supported by the GRRC program of Gyeonggi Province (GRRC‐Ajou2023‐B01 to W.K.) and by Korea Environment Industry & Technology Institute (KEITI) through “Core Technology Development Project for Environmental Diseases Prevention and Management” (RS‐2025‐02214027 to J.W.K.) funded by Korea Ministry of Environment (MOE).

## Conflicts of Interest

The authors declare no conflicts of interest.

## Supporting information




**Supporting File**: advs78313‐sup‐0001‐SuppMat.docx.

## Data Availability

All data associated with this study are present in the main text or the Supporting Information. The RNA‐seq data generated in this study have been deposited in the Gene Expression Omnibus (GEO) database under accession code GEO: GSE276941. *Cnr1*‐floxed mice were obtained from Dr. Josephine M. Egan, IRP/NIA/NIH, through material transfer agreement. Further information and requests for resources and reagents should be directed to Wook Kim (wookkim21@ajou.ac.kr), Eun Kyung Lee (leeek@catholic.ac.kr) and Jung‐Woong Kim (jungkim@cau.ac.kr).

## References

[1] M. A. Lowes , M. Suárez‐Fariñas , and J. G. Krueger , “Immunology of Psoriasis,” Annual Review of Immunology 32, no. 1 (2014): 227–255, 10.1146/annurev-immunol-032713-120225. PMC4229247 24655295

[2] M. Pasparakis , I. Haase , and F. O. Nestle , “Mechanisms Regulating Skin Immunity and Inflammation,” Nature Reviews Immunology 14, no. 5 (2014): 289–301. 10.1038/nri3646 24722477

[3] A. W. Armstrong , A. Blauvelt , K. Callis Duffin , et al., “Psoriasis,” Nature Reviews Disease Primers 11, no. 1 (2025): 45, 10.1038/s41572-025-00630-5. 40571687

[4] C. E. M. Griffiths , A. W. Armstrong , J. E. Gudjonsson , and J. N. W. N. Barker , “Psoriasis,” The Lancet 397, no. 10281 (2021): 1301–1315, 10.1016/S0140-6736(20)32549-6. 33812489

[5] W. H. Boehncke and M. P. Schön , “Psoriasis,” The Lancet 386, no. 9997 (2015): 983–994, 10.1016/S0140-6736(14)61909-7. 26025581

[6] J. E. Greb , A. M. Goldminz , J. T. Elder , et al., “Psoriasis,” Nature Reviews Disease Primers 2, no. 1 (2016): 16082, 10.1038/nrdp.2016.82. 27883001

[7] I. Raposo and T. Torres , “Palmoplantar Psoriasis and Palmoplantar Pustulosis: Current Treatment and Future Prospects,” American Journal of Clinical Dermatology 17, no. 4 (2016): 349–358, 10.1007/s40257-016-0191-7. 27113059

[8] Y. Zhang , Q. Li , E. Rao , et al., “Epidermal Fatty Acid Binding Protein Promotes Skin Inflammation Induced by High‐Fat Diet,” Immunity 42, no. 5 (2015): 953–964, 10.1016/j.immuni.2015.04.016. PMC4440244 25992864

[9] D. A. Mogilenko , J. T. Haas , L. L'homme , et al., “Metabolic and Innate Immune Cues Merge Into a Specific Inflammatory Response via the UPR,” Cell 177, no. 5 (2019): 1201–1216.e19, 10.1016/j.cell.2019.03.018. 31031005

[10] P. Sivasami , C. Elkins , P. P. Diaz‐Saldana , et al., “Obesity‐Induced Dysregulation of Skin‐Resident PPARγ+ Treg Cells Promotes IL‐17A‐Mediated Psoriatic Inflammation,” Immunity 56, no. 8 (2023): 1844–1861.e6, 10.1016/j.immuni.2023.06.021. PMC10527179 37478855

[11] P. Pacher , S. Bátkai , and G. Kunos , “The Endocannabinoid System as an Emerging Target of Pharmacotherapy,” Pharmacological Reviews 58, no. 3 (2006): 389–462, 10.1124/pr.58.3.2. PMC2241751 16968947

[12] S. Engeli , J. Bo?hnke , M. Feldpausch , et al., “Activation of the Peripheral Endocannabinoid System in Human Obesity,” Diabetes 54, no. 10 (2005): 2838–2843, 10.2337/diabetes.54.10.2838. PMC2228268 16186383

[13] C. Silvestri and V. Di Marzo , “The Endocannabinoid System in Energy Homeostasis and the Etiopathology of Metabolic Disorders,” Cell Metabolism 17, no. 4 (2013): 475–490, 10.1016/j.cmet.2013.03.001. 23562074

[14] D. Cota , G. Marsicano , M. Tschöp , et al., “The Endogenous Cannabinoid System Affects Energy Balance via central Orexigenic Drive and Peripheral Lipogenesis,” Journal of Clinical Investigation 112, no. 3 (2003): 423–431, 10.1172/JCI17725. PMC166293 12897210

[15] J. P. Després , A. Golay , and L. Sjöström , “Effects of Rimonabant on Metabolic Risk Factors in Overweight Patients with Dyslipidemia,” New England Journal of Medicine 353, no. 20 (2005): 2121–2134, 10.1056/NEJMoa044537. 16291982

[16] L. F. Van Gaal , A. M. Rissanen , A. J. Scheen , O. Ziegler , S. Rössner , and RIO‐Europe Study Group , “Effects of the Cannabinoid‐1 Receptor Blocker Rimonabant on Weight Reduction and Cardiovascular Risk Factors in Overweight Patients: 1‐Year Experience from the RIO‐Europe Study,” Lancet 365, no. 9468 (2005): 1389–1397. 10.1016/S0140-6736(05)66374-X 15836887

[17] G. Kunos and J. Tam , “The Case for Peripheral CB1 Receptor Blockade in the Treatment of Visceral Obesity and Its Cardiometabolic Complications,” British Journal of Pharmacology 163, no. 7 (2011): 1423–1431, 10.1111/j.1476-5381.2011.01352.x. PMC3165952 21434882

[18] A. King , “Neuropsychiatric Adverse Effects Signal the End of the Line for Rimonabant,” Nature Reviews Cardiology 7, no. 11 (2010): 602, 10.1038/nrcardio.2010.148. 21080606

[19] J. H. Han and W. Kim , “Peripheral CB1R as a Modulator of Metabolic Inflammation,” The FASEB Journal 35, no. 4 (2021): 21232, 10.1096/fj.202001960R. 33715173

[20] R. Cinar , M. R. Iyer , and G. Kunos , “The Therapeutic Potential of Second and Third Generation CB1R Antagonists,” Pharmacology & Therapeutics 208 (2020): 107477, 10.1016/j.pharmthera.2020.107477. PMC8605822 31926199

[21] J. Tam , V. K. Vemuri , J. Liu , et al., “Peripheral CB1 Cannabinoid Receptor Blockade Improves Cardiometabolic Risk in Mouse Models of Obesity,” Journal of Clinical Investigation 120, no. 8 (2010): 2953–2966, 10.1172/JCI42551. PMC2912197 20664173

[22] J. Tam , R. Cinar , J. Liu , et al., “Peripheral Cannabinoid‐1 Receptor Inverse Agonism Reduces Obesity by Reversing Leptin Resistance,” Cell Metabolism 16, no. 2 (2012): 167–179, 10.1016/j.cmet.2012.07.002. PMC3832894 22841573

[23] J. H. Han , H. Shin , J. G. Rho , et al., “Peripheral Cannabinoid 1 Receptor Blockade Mitigates Adipose Tissue Inflammation via NLRP3 Inflammasome in Mouse Models of Obesity,” Diabetes, Obesity and Metabolism 20, no. 9 (2018): 2179–2189, 10.1111/dom.13350. 29740969

[24] J. H. Han , H. Shin , J. Y. Park , et al., “A Novel Peripheral Cannabinoid 1 Receptor Antagonist, AJ5012, Improves Metabolic Outcomes and Suppresses Adipose Tissue Inflammation in Obese Mice,” The FASEB Journal 33, no. 3 (2019): 4314–4326, 10.1096/fj.201801152RR. 30566396

[25] T. Jourdan , G. Godlewski , R. Cinar , et al., “Activation of the Nlrp3 Inflammasome in Infiltrating Macrophages by Endocannabinoids Mediates Beta Cell Loss in Type 2 Diabetes,” Nature Medicine 19, no. 9 (2013): 1132–1140, 10.1038/nm.3265. PMC4050982 23955712

[26] T. Jourdan , G. Szanda , R. Cinar , et al., “Developmental Role of Macrophage Cannabinoid‐1 Receptor Signaling in Type 2 Diabetes,” Diabetes 66, no. 4 (2017): 994–1007, 10.2337/db16-1199. PMC5360301 28082458

[27] A. M. Malfitano , S. Sosa , C. Laezza , M. De Bortoli , A. Tubaro , and M. Bifulco , “Rimonabant Reduces Keratinocyte Viability by Induction of Apoptosis and Exerts Topical Anti‐Inflammatory Activity in Mice,” British Journal of Pharmacology 162, no. 1 (2011): 84–93, 10.1111/j.1476-5381.2010.01047.x. PMC3012408 20880029

[28] C. N. Zawatsky , J. K. Park , J. Abdalla , G. Kunos , M. R. Iyer , and R. Cinar , “Peripheral Hybrid CB1R and iNOS Antagonist MRI‐1867 Displays Anti‐Fibrotic Efficacy in Bleomycin‐Induced Skin Fibrosis,” Frontiers in Endocrinology 12 (2021): 744857, 10.3389/fendo.2021.744857. PMC8505776 34650521

[29] X. Liu , L. Li , J. Jiang , et al., “Role of Type I Cannabinoid Receptor in Sensory Neurons in Psoriasiform Skin Inflammation and Pruritus,” Journal of Investigative Dermatology 143, no. 5 (2023): 812–821.e3, 10.1016/j.jid.2022.10.014. 36410425

[30] P. Anderson , “Post‐Transcriptional Control of Cytokine Production,” Nature Immunology 9, no. 4 (2008): 353–359, 10.1038/ni1584. 18349815

[31] S. Carpenter , E. P. Ricci , B. C. Mercier , M. J. Moore , and K. A. Fitzgerald , “Post‐Transcriptional Regulation of Gene Expression in Innate Immunity,” Nature Reviews Immunology 14, no. 6 (2014): 361–376, 10.1038/nri3682. 24854588

[32] J. Liu and X. Cao , “RBP–RNA Interactions in the Control of Autoimmunity and Autoinflammation,” Cell Research 33, no. 2 (2023): 97–115, 10.1038/s41422-022-00752-5. PMC9892603 36599968

[33] B. Sarkar , Q. Xi , C. He , and R. J. Schneider , “Selective Degradation of AU‐Rich mRNAs Promoted by the p37 AUF1 Protein Isoform,” Molecular and Cellular Biology 23, no. 18 (2003): 6685–6693, 10.1128/MCB.23.18.6685-6693.2003. PMC193711 12944492

[34] E. J. White , G. Brewer , and G. M. Wilson , “Post‐Transcriptional Control of Gene Expression by AUF1: Mechanisms, Physiological Targets, and Regulation,” Biochimica et Biophysica Acta (BBA)—Gene Regulatory Mechanisms 1829, no. 6‐7 (2013): 680–688, 10.1016/j.bbagrm.2012.12.002. PMC3664190 23246978

[35] S. Paschoud , A. M. Dogar , C. Kuntz , B. Grisoni‐Neupert , L. Richman , and L. C. Ku?hn , “Destabilization of Interleukin‐6 mRNA Requires a Putative RNA Stem‐Loop Structure, an AU‐Rich Element, and the RNA‐Binding Protein AUF1,” Molecular and Cellular Biology 26, no. 22 (2006): 8228–8241, 10.1128/MCB.01155-06. PMC1636780 16954375

[36] A. Pautz , K. Linker , S. Altenhöfer , et al., “Similar Regulation of human Inducible Nitric‐Oxide Synthase Expression by Different Isoforms of the RNA‐Binding Protein AUF1,” Journal of Biological Chemistry 284, no. 5 (2009): 2755–2766, 10.1074/jbc.M809314200. 19074427

[37] J. Y. Lu , N. Sadri , and R. J. Schneider , “Endotoxic Shock in AUF1 Knockout Mice Mediated by Failure to Degrade Proinflammatory Cytokine mRNAs,” Genes & Development 20, no. 22 (2006): 3174–3184, 10.1101/gad.1467606. PMC1635151 17085481

[38] N. Sadri and R. J. Schneider , “AUF1/HNRNPD‐Deficient Mice Develop Pruritic Inflammatory Skin Disease,” Journal of Investigative Dermatology 129, no. 3 (2009): 657–670, 10.1038/jid.2008.298. PMC4074411 18830269

[39] I. Ruiz de Azua , G. Mancini , R. K. Srivastava , et al., “Adipocyte Cannabinoid Receptor CB1 Regulates Energy Homeostasis and Alternatively Activated Macrophages,” Journal of Clinical Investigation 127, no. 11 (2017): 4148–4162, 10.1172/JCI83626. PMC5663356 29035280

[40] A. Miranville , A. W. Herling , G. Biemer‐Daub , and M. D. Voss , “Reversal of Inflammation‐Induced Impairment of Glucose Uptake in Adipocytes by Direct Effect of CB1 Antagonism on Adipose Tissue Macrophages,” Obesity 18, no. 12 (2010): 2247–2254, 10.1038/oby.2010.81. 20379144

[41] L. Tian , W. Li , L. Yang , et al., “Cannabinoid Receptor 1 Participates in Liver Inflammation by Promoting M1 Macrophage Polarization via RhoA/NF‐κB p65 and ERK1/2 Pathways, Respectively, in Mouse Liver Fibrogenesis,” Frontiers in Immunology 8 (2017): 1214, 10.3389/fimmu.2017.01214. PMC5625548 29033935

[42] P. Mai , L. Yang , L. Tian , et al., “Endocannabinoid System Contributes to Liver Injury and Inflammation by Activation of Bone Marrow–Derived Monocytes/Macrophages in a CB1‐Dependent Manner,” The Journal of Immunology 195, no. 7 (2015): 3390–3401, 10.4049/jimmunol.1403205. 26320250

[43] T. Jourdan , S. M. Nicoloro , Z. Zhou , et al., “Decreasing CB1 Receptor Signaling in Kupffer Cells Improves Insulin Sensitivity in Obese Mice,” Molecular Metabolism 6, no. 11 (2017): 1517–1528, 10.1016/j.molmet.2017.08.011. PMC5681272 29107297

[44] A. Stratis , “Pathogenic Role for Skin Macrophages in a Mouse Model of Keratinocyte‐Induced Psoriasis‐Like Skin Inflammation,” Journal of Clinical Investigation 116, no. 8 (2006): 2094–2104, 10.1172/JCI27179. PMC1525004 16886058

[45] H. Wang , “Activated Macrophages are Essential in a Murine Model for T Cell‐Mediated Chronic Psoriasiform Skin Inflammation,” Journal of Clinical Investigation 116, no. 8 (2006): 2105–2114, 10.1172/JCI27180. PMC1523400 16886059

[46] M. Rinaldi‐Carmona , F. Barth , M. Héaulme , et al., “Biochemical and Pharmacological Characterisation of SR141716A, the First Potent and Selective Brain Cannabinoid Receptor Antagonist,” Life Sciences 56, no. 23‐24 (1995): 1941–19477, 10.1016/0024-3205(95)00174-5. 7776817

[47] M. Rinaldi‐Carmona , B. Calandra , D. Shire , et al., “Characterization of Two Cloned human CB1 Cannabinoid Receptor Isoforms,” The Journal of Pharmacology and Experimental Therapeutics 278, no. 2 (1996): 871–878, 10.1016/S0022-3565(25)20674-7. 8768742

[48] M. M. van der Lee , M. Blomenröhr , A. A. van der Doelen , et al., “Pharmacological Characterization of Receptor Redistribution and Beta‐Arrestin Recruitment Assays for the Cannabinoid Receptor 1,” Journal of Biomolecular Screening 14, no. 7 (2009): 811–823. 10.1177/1087057109337937 19520790

[49] J. A. Segre , “Epidermal Barrier Formation and Recovery in Skin Disorders,” Journal of Clinical Investigation 116, no. 5 (2006): 1150–1158, 10.1172/JCI28521. PMC1451215 16670755

[50] A. Orsmond , L. Bereza‐Malcolm , T. Lynch , L. March , and M. Xue , “Skin Barrier Dysregulation in Psoriasis,” International Journal of Molecular Sciences 22, no. 19 (2021): 10841, 10.3390/ijms221910841. PMC8509518 34639182

[51] X. Zhou , Y. Chen , L. Cui , Y. Shi , and C. Guo , “Advances in the Pathogenesis of Psoriasis: From Keratinocyte Perspective,” Cell Death & Disease 13, no. 1 (2022): 81, 10.1038/s41419-022-04523-3. PMC8786887 35075118

[52] E. Candi , R. Schmidt , and G. Melino , “The Cornified Envelope: A Model of Cell Death in the Skin,” Nature Reviews Molecular Cell Biology 6, no. 4 (2005): 328–340, 10.1038/nrm1619. 15803139

[53] M. Furuse , M. Hata , K. Furuse , et al., “Claudin‐Based Tight Junctions Are Crucial for the Mammalian Epidermal Barrier,” The Journal of Cell Biology 156, no. 6 (2002): 1099–1111, 10.1083/jcb.200110122. PMC2173463 11889141

[54] R. Leite Dantas , D. Masemann , T. Schied , et al., “Macrophage‐Mediated Psoriasis Can be Suppressed by Regulatory T Lymphocytes,” The Journal of Pathology 240, no. 3 (2016): 366–377, 10.1002/path.4786. 27555499

[55] C.‐H. Lu , C.‐Y. Lai , D.‐W. Yeh , et al., “Involvement of M1 Macrophage Polarization in Endosomal Toll‐Like Receptors Activated Psoriatic Inflammation,” Mediators of Inflammation 2018 (2018): 1–14, 10.1155/2018/3523642. PMC6311781 30647534

[56] W. H. Lee , J. G. I. Rho , Y. Yang , et al., “Hyaluronic Acid Nanoparticles as a Topical Agent for Treating Psoriasis,” ACS Nano 16, no. 12 (2022): 20057–20074, 10.1021/acsnano.2c07843. 36373736

[57] N. van Rooijen and E. Hendrikx , “Liposomes for Specific Depletion of Macrophages from Organs and Tissues,” Methods in Molecular Biology 605 (2010): 189–203. 10.1007/978-1-60327-360-2_13 20072882

[58] X. Chen , G. Deng , K. Chen , Y. Chen , W. Ye , and P. Sun , “Targeting the NLRP3 Inflammasome in Psoriasis,” International Journal of Dermatology 63, no. 7 (2024): 844–851, 10.1111/ijd.17073. 38345734

[59] G. Deng , W. Chen , P. Wang , et al., “Inhibition of NLRP3 Inflammasome‐Mediated Pyroptosis in Macrophage by Cycloastragenol Contributes to Amelioration of Imiquimod‐Induced Psoriasis‐Like Skin Inflammation in Mice,” International Immunopharmacology 74 (2019): 105682, 10.1016/j.intimp.2019.105682. 31203155

[60] N. Irrera , M. Vaccaro , A. Bitto , et al., “BAY 11‐7082 Inhibits the NF‐κB and NLRP3 Inflammasome Pathways and Protects against IMQ‐Induced Psoriasis,” Clinical Science 131, no. 6 (2017): 487–498, 10.1042/CS20160645. 28096316

[61] M. Ciążyńska , I. Olejniczak‐Staruch , D. Sobolewska‐Sztychny , J. Narbutt , M. Skibińska , and A. Lesiak , “The Role of NLRP1, NLRP3, and AIM2 Inflammasomes in Psoriasis,” International Journal of Molecular Sciences 22, no. 11 (2021): 5898. 10.3390/ijms22115898 PMC8198493 34072753

[62] C. Zhang , B. Tang , X. Zheng , et al., “Analysis of the Potential Pyroptosis Mechanism in Psoriasis and Experimental Validation of NLRP3 In Vitro and In Vivo,” International Immunopharmacology 124, no. Pt A (2023): 110811, 10.1016/j.intimp.2023.110811. 37647679

[63] L. Tang and F. Zhou , “Inflammasomes in Common Immune‐Related Skin Diseases,” Frontiers in Immunology 11 (2020): 882, 10.3389/fimmu.2020.00882. PMC7247819 32528469

[64] T. Jourdan , G. Szanda , A. Z. Rosenberg , et al., “Overactive Cannabinoid 1 Receptor in Podocytes Drives Type 2 Diabetic Nephropathy,” Proceedings of the National Academy of Sciences 111, no. 50 (2014): E5420–E5428, 10.1073/pnas.1419901111. PMC4273328 25422468

[65] F. Barutta , R. Mastrocola , S. Bellini , G. Bruno , and G. Gruden , “Cannabinoid Receptors in Diabetic Kidney Disease,” Current Diabetes Reports 18, no. 2 (2018): 9, 10.1007/s11892-018-0975-7. 29399721

[66] S. Udi , L. Hinden , B. Earley , et al., “Proximal Tubular Cannabinoid‐1 Receptor Regulates Obesity‐Induced CKD,” Journal of the American Society of Nephrology 28, no. 12 (2017): 3518–3532, 10.1681/ASN.2016101085. PMC5698062 28860163

[67] T. Jourdan , J. K. Park , Z. V. Varga , et al., “Cannabinoid‐1 Receptor Deletion in Podocytes Mitigates Both Glomerular and Tubular Dysfunction in a Mouse Model of Diabetic Nephropathy,” Diabetes, Obesity and Metabolism 20, no. 3 (2018): 698–708, 10.1111/dom.13150. 29106063

[68] M. Rajesh , S. Bátkai , M. Kechrid , et al., “Cannabinoid 1 Receptor Promotes Cardiac Dysfunction, Oxidative Stress, Inflammation, and Fibrosis in Diabetic Cardiomyopathy,” Diabetes 61, no. 3 (2012): 716–727, 10.2337/db11-0477. PMC3282820 22315315

[69] I. González‐Mariscal , R. A. Montoro , M. E. Doyle , et al., “Absence of Cannabinoid 1 Receptor in Beta Cells Protects Against High‐Fat/High‐Sugar Diet‐Induced Beta Cell Dysfunction and Inflammation in Murine Islets,” Diabetologia 61, no. 6 (2018): 1470–1483, 10.1007/s00125-018-4576-4. PMC6201315 29497784

