# Supplementary material for: Targeting Cannabinoid 1 Receptor in Macrophages Alleviates Psoriatic Skin Inflammation Through the AUF1–NLRP3 Axis
Source: Adv Sci (Weinh). 2026 Oct 14:e78313. Online ahead of print. doi: 10.1002/advs.78313 (PMC13669225; doi:10.1002/advs.78313)
Supplement: Supplementary file 1 [file ADVS-9999-e78313-s001.docx]

Supporting Information

**Targeting Cannabinoid 1 Receptor in Macrophages Alleviates Psoriatic Skin Inflammation through the AUF1–NLRP3 Axis**

**Including:**

Supplemental Materials and Methods

Supplemental Figure S1-23

Supplemental Table S1-S4

**Supplemental Materials and Methods**

**5.1 RIMO synthesis and characterization**

Among the CB1R antagonists used in this study, only RIMO was synthesized in-house. All other CB1R antagonists were obtained from commercial suppliers (Table S1) and used as received. The following procedures describe the synthesis and chemical characterization of RIMO. All reagents and solvents were obtained from commercial suppliers (Sigma-Aldrich, St. Louis, MO, USA/Tokyo Chemical Industry Co., Ltd.,Tokyo, Japan / DAEJUNG, Siheung-si, South Korea / DUKSAN, Ansan-si, South Korea) and used without further purification unless otherwise noted. Reactions were performed in oven-dried glassware under a nitrogen atmosphere. Analytical and purification solvents were laboratory grade and purchased from SK chemicals. Reaction was monitored by thin layer chromatography (TLC, Merck TLC Silica gel 60 F_254_). Visualization was carried out under UV illumination (Spectroline® E-Series UV lamp, 254/365 nm) and by staining with ninhydrin (1.5% ninhydrin (w/v), 97:3 EtOH:AcOH). Flash column chromatography was performed on a Biotage Selekt system using a Biotage Sfär Bio C18 (25 g) cartridge (Biotage, Uppsala, Sweden). Elution was carried out with a Hexane/EtOAc gradient at 80 mL/min. ^1^H and ^13^C NMR spectra were recorded on a 600 MHz spectrometer (JEOL, JNM-ECZ600R). Samples were prepared in CDCl_3_ and DMSO-d6 (ZEOtope®, Rüti, Switzerland)) using 5–20 mg compound in 0.6 mL solvent, and chemical shifts (δ) are reported in parts per million (ppm) relative to internal reference, TMS = 0.00 ppm. ^1^H NMR data are reported as δ (ppm), multiplicity, J (Hz), and integration; multiplicities are abbreviated as s = singlet, d = doublet, dd = doublet of doublet, t = triplet, q = quartet, and m = multiplet. High-performance liquid chromatography (HPLC, Agilent) was performed using an Agilent HPLC system equipped with Eclipse Plus C18 column (4.6 × 50 mm, 5 μm; Agilent Technologies) and LC-MS-grade solvents (Honeywell Charlotte, North Carolina, USA). Compounds were analyzed using a gradient from 10% acetonitrile containing 0.1% formic acid to 100% acetonitrile over 7 min at a flow rate of 1 mL/min. HPLC analysis confirmed a purity of 99% for the final in-house synthesized RIMO preparation. Mass spectra were acquired using an Agilent G6125C single-quadrupole mass spectrometer with electrospray ionization in positive- and negative-ion modes (ESI+ and ESI−). Mass-to-charge ratios are reported as mass-to-charge ratios (*m/z*). The pharmacological properties of in-house synthesized RIMO were characterized using the CB1 Human Cannabinoid GPCR Binding Antagonist Radioligand LeadHunter Assay Assay, CB1 Human Cannabinoid GPCR Cell-Based Antagonist cAMP LeadHunter Assay, and CB1 Human Cannabinoid GPCR Cell-Based Antagonist Arrestin LeadHunter Assay (Eurofins Panlabs, Inc., Taipei, Taiwan). Brain and plasma concentrations of in-house synthesized RIMO were determined 1 h after intraperitoneal administration (10 mg/kg) in mice, as previously described [23, 24]. The brain-to-plasma concentration ratio was calculated by dividing the RIMO concentration in brain tissue by the corresponding plasma concentration.

**5.1.1 Lithium 1-(4-Chlorophenyl)-4-ethoxy-2-methyl-1,3,4-trioxobutan-2-ide (Compound 1)**

1M LiHMDS in THF (97.8 mL, 97.8 mmol, 1.1 equiv.) was added to a solution of 4-chloro propiophenone (15 g, 89.0 mmol, 1.00 equiv.) in MCH (90 mL). The reaction mixture was stirred 30 min at 0℃. Diethyl oxalate (15.8 mL, 115.7 mmol, 1.3 equiv.) was added to reaction mixture and stirred 24 hours at room temp. After stirring, the reaction mixture was filtered and washed by hexane to provide Compound 1 (17.0 g, 69%) as a brown solid. ^1^H NMR (600 MHz, DMSO-*d*_6_) δ 7.28 – 7.25 (m, 2H), 7.23 – 7.21 (m, 2H), 3.71 (d, *J* = 7.4 Hz, 2H), 1.52 (s, 3H), 1.06 (t, *J* = 7.1 Hz, 3H). ^13^C NMR (151 MHz, DMSO-*d*_6_) δ 200.03, 167.84, 162.83, 138.42, 135.81, 130.28, 129.36, 50.59, 50.55, 31.80, 8.50., LC Purity: 99%, MS (ESI-) calc’d for [M-H]^-^ C_13_H_11_ClO_4_^-^ 266.7, found 266.7.

**5.1.2 Ethyl (Z)-4-(4-chlorophenyl)-2-(2-(2,4-dichlorophenyl)hydrazineylidene)-3-methyl-4-oxobutanoate (Compound 2)**

A solution of Compound 1 (13.9 g, 50.87 mmol, 1.00 equiv.) and 2,4-dichloro phenyl hydrazine hydrochloride (10.9 g, 50.87 mmol, 1.00 equiv.) in MeOH (120 mL) was stirred for 48 hours at room temperature. After completion of reaction, the reaction mixture was filtered and washed by MeOH to provide Compound 2 (14.0 g, 64%) as an orange solid. ^1^H NMR (600 MHz, CDCl₃) δ 12.41 (s, 1H), 7.93 – 7.90 (m, 2H), 7.46 (d, *J* = 8.8 Hz, 1H), 7.45 – 7.43 (m, 2H), 7.31 (d, *J* = 2.2 Hz, 1H), 7.19 – 7.17 (m, 1H), 4.72 (q, *J* = 7.0 Hz, 1H), 4.29 – 4.19 (m, 2H), 1.49 (d, *J* = 7.0 Hz, 3H), 1.19 (t, *J* = 7.1 Hz, 3H). ^13^C NMR (151 MHz, CDCl₃) δ 199.14, 162.42, 139.43, 138.70, 134.75, 130.21, 129.90, 129.11, 129.00, 128.14, 126.59, 119.26, 115.30, 61.58, 44.97, 15.15, 14.03., LC Purity: 99%, MS (ESI+) calc’d for [M+H]^+^ C_19_H_18_Cl_3_N_2_O_3_^+^ 428.7, found 428.7.

**5.1.3 Ethyl 5-(4-chlorophenyl)-1-(2,4-dichlorophenyl)-4-methyl-1H-pyrazole-3-carboxylate (Compound 3)**

A solution of Compound 2 (14.0 g, 5.96 mmol, 1.00 equiv.) in AcOH (120 mL) was stirred for 24 hours with reflux condition. After stirring, the reaction mixture was cooled to room temperature and added ice until the precipitate is formed. The precipitate was filtered and washed by H_2_O to provide compound Compound 3 (10.0 g, 74%) as an orange solid. ^1^H NMR (600 MHz, CDCl₃) δ 7.38 (d, *J* = 2.2 Hz, 1H), 7.35 (d, *J* = 8.5 Hz, 1H), 7.32 – 7.30 (m, 2H), 7.08 – 7.06 (m, 2H), 4.46 (q, *J* = 7.1 Hz, 2H), 2.34 (s, 3H), 1.43 (t, *J* = 7.1 Hz, 3H). ^13^C NMR (151 MHz, CDCl₃) δ 162.73, 142.97, 142.93, 136.06, 135.94, 135.02, 133.03, 130.97, 130.86, 130.09, 128.92, 127.86, 127.08, 119.14, 60.97, 14.47, 9.73., LC Purity: 99%, MS (ESI+) calc’d for [M+H]^+^ C_19_H_16_Cl_3_N_2_O_2_^+^ 410.7 found 410.7.

**5.1.4 5-(4-chlorophenyl)-1-(2,4-dichlorophenyl)-4-methyl-1H-pyrazole-3-carboxylic acid (Compound 4)**

2.5 N NaOH solution (3.6 mL) was added to a solution of Compound 3 (1.40 g, 3.596 mmol, 1 equiv.) in THF (3.6 mL). The reaction mixture was stirred 8 hours at 100°C with reflux condition. After stirring, 1N HCl was added to a reaction mixture until pH 1. The mixture was extracted with EtOAc and washed by H_2_O. The organic layer was dried over sodium sulfate, filtered, and concentrated. The concentrate was recrystallized to provide Compound 4 (0.79 g, 58%) as a white solid. ^1^H NMR (600 MHz, DMSO-*d*_6_) δ 7.76 (d, *J* = 2.3 Hz, 1H), 7.68 (d, *J* = 8.5 Hz, 1H), 7.55 (dd, *J* = 8.5, 2.4 Hz, 1H), 7.43 (d, *J* = 8.6 Hz, 2H), 7.19 (d, *J* = 8.5 Hz, 2H), 3.28 (s, 3H). ^13^C NMR (151 MHz, DMSO-*d*_6_) δ 164.19, 143.40, 142.95, 136.31, 135.67, 134.36, 132.48, 132.28, 131.87, 130.19, 129.31, 128.92, 127.62, 118.49, 10.00., LC Purity: 99%, MS (ESI+) calc’d for [M+H]^+^ C_17_H_11_Cl_3_N_2_O_2_^+^ 381.0 found 381.0

**5.1.5 5-(4-chlorophenyl)-1-(2,4-dichlorophenyl)-4-methyl-N-(piperidin-1-yl)-1H-pyrazole-3-carboxamide (Compound 5)**

Oxalyl chloride (533 μL, 6.210 mmol, 3 equiv.) was added to a solution of Compound 4 (0.79 g, 2.070 mmol, 1 equiv.) in DCM (10 mL). Add a drop of DMF to initiate the reaction. The reaction mixture was stirred 4 hours at 50°C with reflux condition. After stirring, the reaction mixture was concentrated and dissolved in DCM. Dissolved mixture was added to a solution of 1-Aminopiperidin (670 μL, 6.210 mmol, 3 equiv.) in DCM (10 mL). The reaction mixture was stirred 2 hours at room temperature with TLC monitoring. The reaction mixture was extracted with EtOAc and washed by H_2_O. The organic layer was dried over sodium sulfate, filtered, and concentrated. The concentrate was purified by flash column chromatography (1:99 to 30:70; EtOAc/Hexane) to provide Compound 5 (543 mg, 57 %) as white solid. ^1^H NMR (600 MHz, DMSO-*d*_6_) δ 7.80 – 7.74 (m, 2H), 7.58 (dd, *J* = 8.5, 2.3 Hz, 1H), 7.47 – 7.42 (m, 2H), 7.26 – 7.18 (m, 2H), 3.30 (s, 4H), 2.23 (s, 3H), 1.78 (t, *J* = 5.9 Hz, 4H), 1.43 (s, 2H). ^13^C NMR (151 MHz, DMSO-*d*_6_) δ 160.39, 143.53, 135.97, 135.94, 134.60, 132.53, 132.51, 131.88, 130.21, 129.39, 129.01, 127.20, 118.26, 56.65, 23.78, 21.87, 9.54., LC Purity: 99%, MS (ESI+) calc’d for [M+H]^+^ C_22_H_21_Cl_3_N_4_O^+^ 462.1 found 462.1.

**Supplemental Figure S1.** CB1R expression increases with disease severity during the progression of IMQ-induced psoriasis-like inflammation. (A) Representative gross images of shaved dorsal skin (top) and spleens (bottom) on day 4. (B) Clinical PASI score (erythema, scaling, and thickness) of dorsal skin during the experimental period (*n* = 3 mice per group). (C) Spleen weights of the indicated groups (*n* = 3 per group). (D) Representative H&E images of dorsal skin sections and quantification of epidermal thickness (*n* = 3 mice per group). Scale bar, 100 μm. (E) Levels of TNF‑α, IL‑1β, IL-17A, and IL-23p19 in dorsal skin tissues from IMQ–inflamed mice (*n* = 12 mice per group). (F) Representative western blots of CB1R in dorsal skin from IMQ–inflamed mice; β‑actin, loading control. Densitometric quantification of CB1R normalized to β‑actin is shown on right (n = 3 mice per group). Data are presented as mean ± SEM. * *P* < 0.05; ** *P* < 0.01; *** *P* < 0.001, analyzed by one-way ANOVA with Dunnett’s multiple comparisons test (B−D, F) or unpaired two-tailed Student’s *t* test (G).

**Supplemental Figure S2.** Validation of CB1R abundance in macrophages using a FAM-conjugated anti-CB1R antibodies (CB1R–FAM). (A) Flow cytometric gating strategy for analyzing CB1R‑FAM binding in F4/80^+^CD11b^+^ BMDMs from WT, M-*Cnr1*^-/-^, and *Cnr1*^-/-^ mice. (B) Flow‑cytometric analysis and quantification of CB1R‑FAM binding in F4/80^+^CD11b^+^ BMDMs from WT, M-*Cnr1*^-/-^, and *Cnr1*^-/-^ mice. (C) Confocal immunofluorescence of F4/80^+^CD11b^+^ BMDMs from WT, M-*Cnr1*^-/-^, and *Cnr1*^-/-^ mice showing CB1R-FAM (green) and nuclei (DAPI, blue). Scale bars, 100 μm.

**Supplemental Figure S3.** Assessment of *Cnr1* gene knockout in *Cnr1^-/-^* and M-*Cnr1^-/-^* mice. (A) Schematic of the floxed *Cnr1* allele (*Cnr1*^flox^; exon 2 flanked by LoxP sites) and the recombined null allele (*Cnr1*^Δ^) after Cre‑mediated excision. Black bars, homology arms; white triangles, LoxP sites; half‑arrows, PCR primers (LOX1, NDL1, NDL2, SDL2). (B) Genomic PCR of BMDMs from WT and myeloid‑specific *Cnr1* knockout (M-*Cnr1*^-/-^) mice. Exon 2 excision was detected with the LOX1/NDL2 primer set (397 bp, recombined allele). The LOX1/SDL2 set distinguishes floxed (*Cnr1*^flox^, 487 bp) and WT (427 bp) alleles and serves as a genotyping control. (C) *Cnr1* mRNA expression in BMDMs and brain across genotypes (WT, *Cnr1*^-/-^, M-*Cnr1*^-/-^), measured by PCR. (D) Confocal immunofluorescence of brain sections showing CB1R (red) and nuclei (DAPI, blue) in WT, *Cnr1*^-/-^, and M-*Cnr1*^-/-^ mice. Scale bars, 100 μm. (E) Western blots of CB1R in BMDMs and BMDCs from the indicated genotypes; β‑actin, loading control.

**Supplemental Figure S4.** Flow cytometric gating strategy and immune-cell profiles in WT, *Cnr1*^-/-^, and M-*Cnr1*^-/-^ mice. (A–C) Flow-cytometry plots of splenic lymphocyte subsets from WT, *Cnr1*^-/-^, and M-*Cnr1*^-/-^ mice. Live singlets in single‑cell suspensions were gated, and the frequencies of CD45^+^ CD3^-^ CD19^+^ B cells (A), CD45^+^CD3^+^CD4^+^ T helper cells (B), and CD45^+^CD3^+^CD4^+^ cytotoxic T cells (C) were determined. (D) Flow-cytometry plots of peritoneal macrophages from peritoneal cavity of WT, *Cnr1*^-/-^, and M-*Cnr1*^-/-^ mice. Live singlets in single‑cell suspensions were gated, and the frequencies of CD45^+^F4/80^+^CD11b^+^ macrophages were determined.

**Supplemental Figure S5.** Flow cytometric gating strategy for the analysis of CD45⁺F4/80⁺CD11b⁺ macrophages in dorsal and ear skin from control and IMQ- or rIL-23-inflamed mice.

**Supplemental Figure S6.** Gross images of IMQ– and rIL‑23–inflamed mouse skin at day 5 prior to flow‑cytometric analysis. (A and B) Skin images of control and IMQ–inflamed mice (A) and from control and rIL‑23–inflamed mice (B). Images were taken immediately before tissue dissociation for the flow‑cytometric experiments shown in Fig. 1C (*n* = 5 mice per group).

**Supplemental Figure S7.** Confocal immunofluorescence of dorsal and ear skin sections showing CB1R (green), F4/80 (red), and nuclei (DAPI, blue) in IMQ– or rIL‑23–inflamed *Cnr1*^-/-^ mice. Scale bars, 100 μm.

**Supplemental Figure S8.** RIMO suppresses pro‑inflammatory gene expression in the IMQ–induced psoriasis model. (A–C) *Casp1* (A), *Il6* (B), and *Ptgs2* (C) mRNA levels in dorsal skin from the groups shown in Fig. 2f, measured by qPCR and normalized to *Gapdh* (*n* = 5–6 mice per group). (D) Representative images and quantification of immunohistochemical staining for TNF‑α and IL‑1β in dorsal skin sections from each group (*n* = 4–5 mice per group). Scale bars, 100 μm. Data are presented as mean ± SEM. *** *P* < 0.001, analyzed by one-way ANOVA with Tukey’s multiple comparisons test (A–D).

**Supplemental Figure S9.** Flow cytometric gating strategy for the analysis of immune cell subsets in dorsal skin from control and IMQ-inflamed mice, related to Figures 2H, 2I, S12G, and S17.

**Supplemental Figure S10.** Anti-psoriatic effects of first- and second-generation CB1R antagonists in the IMQ–induced psoriasis model. (A) Experimental design. First- and second-generation CB1R antagonists (10 mg/kg) or vehicle was administered intraperitoneally 30 min prior to topical application of Aldara cream (5% IMQ) on days 1–4 using a preventive treatment protocol. (B) Representative gross images of shaved dorsal skin on day 5. (C) Clinical PASI score of dorsal skin over five days for the mice shown in (B) (*n* = 5–10 mice per group). (D) Representative H&E images (top) and PCNA immunohistochemistry (bottom) of dorsal skin sections on day 5 (*n* = 5 mice per group). Epidermal thickness and the percentage of Ki-67-positive cells in the epidermis were quantified and are shown below. Scale bars, 100 μm. (E) Representative images (top) and quantification (bottom) of immunohistochemical staining for TNF‑α, IL‑1β, IL-17A, IL-23p19, and CD68 in dorsal skin sections from each group (*n* = 5 mice per group). Scale bars, 100 μm. Data are presented as mean ± SEM. * *P* < 0.05; ** *P* < 0.01; *** *P* < 0.001, analyzed by one-way ANOVA with Tukey’s multiple comparisons test (D and E) or two-way ANOVA followed by Tukey’s multiple comparisons test (C).

**Supplemental Figure S11**. RIMO protects against skin‑barrier disruption in the IMQ–induced psoriasis model. (A and B) Representative images of immunohistochemical staining for loricrin and involucrin in dorsal skin sections from control and IMQ‑inflamed mice treated with vehicle or RIMO (10 mg/kg) (A), and IMQ–inflamed WT, *Cnr1^-/-^*, and M-*Cnr1^-/-^* mice (B). Scale bars, 100 μm. (C) *Lor*, *Defb3*, *S100a9*, and *Il6* mRNA levels in CD45^-^ cells isolated from dorsal skins of the groups shown in Fig. 2H. Cells from five mice per group were pooled, flow-sorted, and analyzed by qPCR; values were normalized to *Gapdh*. (D) Western blots of Claudin-1 and S100A9 in dorsal skins of IMQ–inflamed mice treated with vehicle or RIMO; β‑actin, loading control. Densitometric quantification normalized to β‑actin is shown below (*n* = 3 mice per group). (E) Assessment of skin‑barrier function by transcutaneous permeability of FITC–dextran. On day 5, 4 kDa FITC–dextran (5 mg/cm^2^) was transcutaneously applied to shaved dorsal skin for 6 h, and circulating FITC–dextran levels in the blood were measured (*n* = 4–5 mice per group). Data are presented as mean ± SEM. * *P* < 0.05; *** *P* < 0.001, analyzed by one-way ANOVA with Tukey’s multiple comparisons test (D and E).

**Supplemental Figure S12.** Post-onset administration of RIMO improves psoriatic symptoms. (A) Experimental design. C57BL/6 mice received topical vehicle or Aldara cream (5% imiquimod, IMQ) once daily on days 1–4. On days 3 and 4, RIMO (10 mg/kg) or vehicle was administered intraperitoneally (i.p.) 30 min prior to IMQ application using a therapeutic treatment protocol. (B) Representative gross images of shaved dorsal skin (top) and spleens (bottom) on day 5. (C) Clinical PASI score of dorsal skin over five days for the mice shown in (B, top) (*n* = 9 mice per group). (D) Spleen weight for the mice shown in (B, bottom) (*n* = 5 per group). (E) Representative H&E images (top) and PCNA immunohistochemistry (bottom) of dorsal skin sections on day 5 (*n* = 5 mice per group). Epidermal thickness and the percentage of PCNA-positive epidermal cells were quantified and are shown below. Scale bars, 100 μm. (F) Representative images (top) and quantification (bottom) of immunohistochemical staining for IL‑1β and CD68 in dorsal skin sections from each group (*n* = 5 mice per group). Scale bars, 100 μm. (G) Representative flow-cytometry plots of immune-cell subsets in dorsal skins from each group. Live singlets in single‑cell suspensions were gated, and the frequencies of CD45^+^ immune cells, CD45^+^CD3^+^ T cells, CD45^+^CD11c^+^ dendritic cells, CD45^+^Ly6G^+^ neutrophils, and CD45^+^F4/80^+^CD11b^+^CD86^+^ macrophages were determined. Data are presented as mean ± SEM. * *P* < 0.05; *** *P* < 0.001, analyzed by one-way ANOVA with Tukey’s multiple comparisons test (D–F) or two-way ANOVA followed by Tukey’s multiple comparisons test (C).

**Supplemental Figure S13.** Clodronate-mediated depletion of macrophages in control and IMQ–inflamed mice. (A) Flow-cytometry plots of F4/80^+^ CD11b^+^ peritoneal macrophages from peritoneal cavity of normal mice administered intraperitoneally with control or clodronate liposomes (50 mg). (B) Representative images (left) and quantification (right) of immunohistochemical staining for CD68 in skin sections from each group (*n* = 5–6 mice per group). Control and clodronate liposomes (50 mg) were administered intraperitoneally on day 0 and 3, as in Fig. 3a. Scale bars, 100 μm. (C) Representative images (left) and quantification (right) of immunohistochemical staining for IL‑17A, IL-23p19, and CD4 in dorsal skin sections from each group (n = 5–6 mice per group). Scale bars, 100 μm. Data are presented as mean ± SEM. *** *P* < 0.001, analyzed by two-way ANOVA followed by Tukey’s multiple comparisons test (B and C).

**Supplemental Figure S14.** Adoptive transfer of WT, but not CB1R-deficient, macrophages restores IMQ-induced psoriasis-like phenotypes in macrophage-depleted mice. (A) Experimental design. Macrophages were depleted by a single subcutaneous injection of clodronate liposomes into the dorsal skin 2 days before the first IMQ application. On the first day of IMQ treatment, Far Red-labeled WT or *Cnr1*^−/−^ BMDMs were administered subcutaneously into the dorsal region 4 h before IMQ application, followed by daily topical application of IMQ for four consecutive days. (B) Representative fluorescence images showing the localization of adoptively transferred, Far Red-labeled WT or *Cnr1*^−/−^ BMDMs (green) in dorsal skin on day 5. Nuclei were counterstained with DAPI (blue). Scale bars, 100 μm. (C) Representative gross images of shaved dorsal skin from the indicated groups on day 5. (D) Clinical PASI score of dorsal skin assessed during the experimental period (*n* = 5 mice per group). (E) Representative H&E images (left) of dorsal skin sections and quantification of epidermal thickness (right) from the indicated groups on day 5 (*n* = 5 mice per group). Scale bars, 100 μm. (F) Representative images (left) and quantification (right) of immunohistochemical staining for TNF-α and IL-1β in dorsal skin sections from each group (n = 5 mice per group). Scale bars, 100 μm. Data are presented as mean ± SEM. * *P* < 0.05; ** *P* < 0.01; *** *P* < 0.001, analyzed by one-way ANOVA followed by Tukey’s multiple comparisons test (D–F).

**Supplemental Figure S15.** Representative images (left) and quantification (right) of immunohistochemical staining for TNF‑α, IL‑17A, IL-23p19, and CD68 in dorsal skin sections from each group (n = 5 mice per group). Scale bars, 100 μm. Data are presented as mean ± SEM. ** *P* < 0.01; *** *P* < 0.001, analyzed by two-way ANOVA with Tukey’s multiple comparison test.

**Supplemental Figure S16.** Myeloid cell–specific CB1R deficiency ameliorates rIL-23–induced psoriasis-like phenotypes *in vivo*. (A) Representative gross images of the ear (inner and outer surfaces) on day 9. WT, *Cnr1^-/-^*, and M-*Cnr1^-/-^* mice received intradermal vehicle or rIL-23 (500 ng) once daily on days 1–8. (B) Representative images (top) and weights (bottom) of spleens from the mice shown in (A) (*n* = 4–5 per group). (C) Representative H&E images (top) and Ki-67 immunohistochemistry (bottom) of ear sections on day 9. Epidermal thickness and the percentage of Ki-67-positive epidermal cells were quantified and are shown at right (*n* = 4–5 mice per group). Scale bars, 100 μm. (D) Representative images (left) and quantification (right) of immunohistochemical staining for TNF‑α, IL‑1β, and CD68 in ear sections from each group (*n* = 4–5 mice per group). Scale bars, 100 μm. Data are presented as mean ± SEM. * *P* < 0.05; ** *P* < 0.01; *** *P* < 0.001, analyzed by one-way ANOVA with Tukey’s multiple comparisons test (B) or two-way ANOVA followed by Tukey’s multiple comparisons test (C and D).

**Supplemental Figure S17.** Effects of RIMO on immune cell composition in the ear of rIL-23-inflamed mice. Flow‑cytometric analysis of single‑cell suspensions from ear skin of control and rIL‑23–inflamed mice treated with vehicle or RIMO (10 mg/kg) using a preventive treatment protocol. Live singlets in single‑cell suspensions were gated, and the frequencies of CD45^+^ immune cells, CD45^+^CD3^+^ T cells, CD45^+^CD11c^+^ dendritic cells, CD45^+^Ly6G^+^ neutrophils, and CD45^+^F4/80^+^CD11b^+^ macrophages were determined. Numbers adjacent to outlined areas indicate the percentage of CD45^+^ cells among live cells or the percentage of each indicated subset among CD45^+^‑gated cells.

**Supplemental Figure S18.** Blockade of CB1R suppresses M1 polarization of macrophages. (A) *Il1b*, *Tnf*, *Il6*, *Il23a*, and *Il12b* mRNA levels in BMDMs from WT and *Cnr1*^-/-^ mice after stimulation with LPS (10 ng/ml), measured by qPCR and normalized to *Gapdh* (*n* = 5). (B) *Il1b*, *Tnf*, *Il6*, *Il23a*, and *Ptgs2* mRNA levels in RAW264.7 cells stimulated with LPS (100 ng/ml) in the presence or absence of RIMO (5 μM), measured by qPCR and normalized to *Gapdh* (*n* = 3). (C) *Il1b*, *Tnf*, *Il6*, *Il23a*, and *Il12b* mRNA levels in BMDMs from WT and *Cnr1*^-/-^ mice after stimulation with IMQ (1 g/ml), measured by qPCR and normalized to *Gapdh* (*n* = 5). (D) Flow cytometric gating strategy for analyzing CD86 expression in F4/80^+^CD11b^+^ BMDMs from WT and *Cnr1*^-/-^ mice. (E) Flow‑cytometric analysis of CD86 in BMDMs from WT and *Cnr1*^-/-^ mice after LPS (10 ng/ml) stimulation in the presence or absence of RIMO (5 μM). The percentage of CD86^+^ cells determined by flow cytometry is shown at right. (F) Flow‑cytometric analysis of CD86 in WT BMDMs after LPS (10 ng/ml) or IMQ (1 µg/ml) stimulation in the presence or absence of RIMO (5 μM). Mean fluorescence intensities (MFIs) determined by flow cytometry is shown at right (*n* = 3). Data are presented as mean ± SEM. * *P* < 0.05; ** *P* < 0.01; *** *P* < 0.001, analyzed by one-way ANOVA with Tukey’s multiple comparisons test (B and E) or two-way ANOVA followed by Tukey’s multiple comparisons test (A and C).

**Supplemental Figure S19.** Differential expression and AU-rich element–mediated regulation of *Nlrp3* in CD45^+^ immune cells from IMQ– and rIL-23–inflamed mice. (A–D) Volcano plots showing differential gene expression in sorted CD45^+/+^ skin immune cells (as in Fig. 5) for the comparisons IMQ vs Sham (A), rIL‑23 vs Sham (B), RIMO vs IMQ (C), and RIMO vs rIL‑23 (D). Axes depict log2 fold change versus −log10 *P* (significance thresholds as indicated); selected genes are labeled, including *Nlrp3*. (E) KEGG pathway enrichment of genes down-regulated in IMQ– or rIL‑23–inflamed groups and reversed toward control levels by RIMO (significance thresholds as indicated). (F) Schematic of the mouse *Nlrp3* gene highlighting AU‑rich region (AU) within the 3′ UTR proposed to mediate mRNA destabilization.

**Supplemental Figure S20.** Modulation of AUF1 and NLRP3 expression in macrophages by CB1R blockade. (A) *AUF1* (*HNRNPD*) mRNA expression in non-lesional (*n* = 67) and lesional skin (*n* = 125) of patients with psoriasis from RNA-seq dataset GSE106992. (B and C) *Auf1* mRNA levels in RAW264.7 cells (B) and BMDMs (C) stimulated with AEA (2 μM) in the presence or absence of RIMO (1 μM), measured by qPCR and normalized to *Gapdh* (*n* = 3). (D) *NLRP3* mRNA expression in non‑lesional (n = 67) and lesional skin (n = 125) from the dataset in (A). (E) *Nlrp3* mRNA levels in BMDMs from WT and *Cnr1^-/-^* mice after stimulation with LPS (10 ng/ml), measured by qPCR and normalized to *Gapdh* (*n* = 5). (F) *Nlrp3* mRNA levels in BMDMs cells stimulated with LPS (100 ng/ml) in the presence or absence of RIMO (1 μM), measured by qPCR and normalized to *Gapdh* (*n* = 3). Data are presented as mean ± SEM. * *P* < 0.05; ** *P* < 0.01; *** *P* < 0.001, analyzed by unpaired two-tailed *t*-test with Welch’s correction (A and E) or one-way ANOVA with Tukey’s multiple comparisons test (B–D) or two-way ANOVA followed by Tukey’s multiple comparisons test (F).

**Supplemental Figure S21.** Effects of RIMO in IMQ–inflamed WT and *Nlrp3^-/-^* mice. (A) Representative H&E images (left) and epidermal thickness (right) of dorsal skin sections from WT and *Nlrp3^-/-^* mice on day 5 (*n* = 4–5 mice per group). RIMO (10 mg/kg) or vehicle was administered intraperitoneally 30 min prior to topical application of Aldara cream (5% IMQ) on days 1–4 using a preventive treatment protocol. Scale bars, 100 μm. (B) Representative images (left) and quantification (right) of immunohistochemical staining for IL‑1β, IL‑17A, IL-23p19, and CD68 in dorsal skin sections from each group (*n* = 4–5 mice per group). Scale bars, 100 μm. Data are presented as mean ± SEM. * *P* < 0.05; ** *P* < 0.01; *** *P* < 0.001, analyzed by two-way ANOVA followed by Tukey’s multiple comparisons test (A and B).

**Supplemental Figure S22.** Evaluation of adenovirus-mediated AUF1 (Ad-*Auf1*) overexpression in normal mice. (A) Experimental design. Ad-Ctrl or Ad-*Auf1* was injected intradermally into the ear on day 1. RIMO (10 mg/kg) or vehicle was administered intraperitoneally 30 min prior to intradermal injection of vehicle or rIL-23 (500 ng) on days 9–16. (B) Representative images of immunohistochemical staining for AUF1 in dorsal skin sections from normal mice infected with Ad-*Auf1*. Scale bars, 100 μm. (C) Body weight change for the mice shown in (B) (*n* =10 per group). (D) Representative H&E images of major organ sections from the mice shown in (B) on day 8.

**Supplemental Figure S23.** Effects of RIMO and AUF1 overexpression on the rIL-23–inflamed WT and *Nlrp3^-/-^* mice. (A–D) Representative images (left) and quantification (right) of immunohistochemical staining for TNF‑α (A), IL‑1β (B), IL-17A (C), and CD68 (D) in ear sections from the mice shown in Fig. 7 on day 17 (*n* = 7–10 mice per group). Scale bars, 100 μm.

Data are presented as mean ± SEM. ** *P* < 0.01; *** *P* < 0.001, analyzed by two-way ANOVA followed by Tukey’s multiple comparisons test (A–D).

**Supplemental Tables**

**Supplemental Table S1.** Information on materials used in this study, including antibodies and chemical reagents.

| ANTIBODIES | SOURCE | IDENTIFIER |
| --- | --- | --- |
| AUF1 | EMD Millipore | Cat# 07-260; RRID: AB_2117338 |
| Anti-Mouse CD3ε (FITC) | Biolegend | Cat# 53-0031-82;  RRID: AB_469889 |
| Anti-Mouse CD8a (PE) | Invitrogen™ | Cat# 12-0081-82;  RRID: AB_465530 |
| Anti-Mouse CD11b (FITC) | Invitrogen™ | Cat# 53-0112-82;  RRID: AB_469901 |
| Anti-Mouse CD11c (Percp) | BioLegend | Cat# 117326; RRID: AB_2129643 |
| Anti-Human CD19 (PE) | Invitrogen™ | Cat# 12-0199-42;  RRID: AB_1834376 |
| Anti-Mouse CD45 (APC-Cy7) | Invitrogen™ | Cat# A15395; RRID: AB_2534409 |
| Anti-Mouse CD45 (PE) | Invitrogen™ | Cat# 12-0451-83;  RRID: AB_465669 |
| Anti-Mouse CD86 (APC) | Invitrogen™ | Cat# 17-0862-81;  RRID: AB_469419 |
| Anti-Mouse CD206 (PE) | Invitrogen™ | Cat# 141706; RRID: AB_2637420 |
| Anti-Mouse CD206 (APC) | Invitrogen™ | Cat# 17-2061-82;  RRID: AB_2637420 |
| Anti-Mouse F4/80 (PE-Cy7) | Invitrogen™ | Cat# 25-4801-82;  RRID: AB_469653 |
| Anti-Mouse Ly6G (PE-Cy7) | Invitrogen™ | Cat# 25-5931-82;  RRID: AB_469663 |
| Alexa Fluor^TM^ 488  Goat Anti-Rat IgG (H+L) | Invitrogen™ | Cat# A11006; RRID: AB_2534074 |
| Alexa Fluor^TM^ 546  Goat Anti-Mouse IgG (H+L) | Invitrogen™ | Cat# A11030; RRID: AB_2737024 |
| Alexa Fluor^TM^ 546  Goat Anti-Rat IgG (H+L) | Invitrogen™ | Cat# A11081; RRID: AB_2534125 |
| Alexa Fluor^TM^ 633  Goat Anti-Rabbit IgG (H+L) | Invitrogen™ | Cat# A21070; RRID: AB_2535731 |
| β-actin | Abcam | Cat# Ab8226; RRID: AB_306371 |
|  | Genetex | Cat# GTX629630;  RRID: AB_2728646 |
| CB1R | Cell Signaling | Cat# 93815S; RRID: AB_2756361 |
|  | Thermo Scientific | Cat# PA1-743; RRID: AB_2245118 |
| CD4 | R&D Systems | Cat# MAB554; RRID: AB_358492 |
| CD68 | R&D Systems | Cat# MAB 101141-100;  RRID: AB_3657120 |
|  | Cell Signaling | Cat# 27344S; RRID: AB_2799882 |
| CellTrace™ Far Red | Invitrogen™ | Cat# C34564 |
| Claudin-1 | Abcam | Cat# Ab15098; RRID: AB_301644 |
| Cleaved Caspase-1 (ASP296) | Cell Signaling | Cat# 89332S; RRID: AB_2923067 |
| F4/80 | Novus Biologicals | Cat# NB600-404;  RRID: AB_10003219 |
| FAM-conjugated CB1R (FAM) | Ajou University | Conjugated CB1R Ab (PA1-743) |
| FcγR-blocking monoclonal antibody (Purified Rat Anti-Mouse CD16/CD32) | BD Biosciences | Cat# BD553142 |
| Goat Anti-Mouse IgG  (H+L)-HRP conjugate | Bio-Rad | Cat# 1721011; RRID: AB_2617113 |
| Goat Anti-Mouse IgG Antibody (H+L), Biotinylated, R.T.U. | VectorLabs | Cat# BR172-1011 |
| Goat Anti-Mouse IgG  Antibody, Peroxidase Conjugated, (H+L) | Sigma-Aldrich | Cat# AP124P; RRID: AB_90456 |
| Goat Anti-Rabbit IgG  (H+L)-HRP conjugate | Bio-Rad | Cat# 1706515; RRID: AB_11125142 |
| Goat Anti-Rabbit IgG  Antibody, Peroxidase Conjugated | Sigma-Aldrich | Cat# AP132P; RRID: AB_90264 |
| Goat Anti-Rabbit IgG Antibody (H+L), Biotinylated, R.T.U. | VectorLabs | Cat# BP9100-50 |
| Goat Anti-Rat IgG Antibody (H+L), Biotinylated, R.T.U. | VectorLabs | Cat# BP-9400-50 |
| IL-1β | Novus Biologicals | Cat# NB600-633;  RRID: AB_577977 |
| IL-17A | Novus Biologicals | Cat# NBP1-72027;  RRID: AB_11034713 |
| IL-23 | Novus Biologicals | Cat# NBP1-76697;  RRID: AB_11027703 |
| Involucrin | BioLegend | Cat# 924401; RRID: AB_2565452 |
| Ki-67 | Cell Signaling | Cat# 12202S; RRID: AB_2620142 |
| Loricrin | Abcam | Cat# Ab85679; RRID: AB_2134912 |
| NLRP3 | Thermo Scientific | Cat# MA5-23919;  RRID: AB_2605972 |
|  | Adipogen | Cat# AG-20B-0014-C100;  RRID: AB_2490202 |
| Normal Rabbit IgG | Sigma-Aldrich | Cat# 12-370; RRID: AB_145841 |
| Mouse monoclonal anti-GFP | Santa Cruz | Cat# SC-9996; RRID: AB_627695 |
| PCNA | Santa Cruz | Cat# SC-56; RRID: AB_628110 |
| S100A9 | Novus Biologicals | Cat# NB110-89726;  RRID: AB_1217846 |
| TNF-α | Novus Biologicals | Cat# NBP1-19532;  RRID: AB_1643202 |
| Biological samples | | |
| Human skin paraffin sections | Seoul St. Mary’s Hospital, The Catholic University of Korea | (Please see details in STAR methods section) |
| Chemicals, peptides, and recombinant proteins | | |
| 1-Aminopiperidin | TCI | Cat# A2241 |
| 1 M LiHMDS in THF | Sigma-Aldrich | Cat# 225770 |
| 2,4-Dichlorophenylhydrazine hydrochloride | TCI | Cat# D2433 |
| 4′-Chloropropiophenone | Sigma-Aldrich | Cat# C69209 |
| 5-carboxyfluorescein-C2TCO-NHS linker | Ajou University | N/A |
| ACEA | TOCRIS | Cat# 1319 |
| Acetic aicd | TCI | Cat# A2035 |
| AM251 | Sigma-Aldrich | Cat# A6226 |
| AM6545 | Sigma-Aldrich | Cat# A1987 |
| Anandamide (AEA) | TOCRIS | Cat# 1339 |
| BPRCB1184 | MCE | Cat# HY-12790 |
| Clodronate liposome | FormuMax Scientific | Cat# F70101C-NC |
| CP55,940 | Cayman Chemicals | Cat# 83003-12-7 |
| Diethyl oxalate | Sigma-Aldrich | Cat# 135364 |
| FITC-dextran | Sigma-Aldrich | Cat# FD4 |
| GM-CSF | Abcam | Cat# ab9742 |
| Hydrochloric acid | DUKSAN | Cat# 2065 |
| IHC antigen retrieval solution | Invitrogen™ | Cat# 00-4956-58 |
| Imiquimode (IMQ) | Invivogen | Cat# R837 |
| Imiquimod cream (5%) | 3M Pharmaceuticals | Cat# Aldara |
| JD5037 | MCE | Cat# HY-18697 |
| Lipopolysaccaride (LPS) | Sigma-Aldrich | Cat# L5293 |
| M-CSF | Sino Biological | Cat# 51112-MNAH |
| Mouse recombinant IL-4 | Novoprotein | Cat# CK15 |
| Mouse recombinant IL-23 | eBioscience | Cat# 14-8231-63 |
| Oxalyl chloride | TCI | Cat# 00082 |
| Rimonabant | Ajou University | N/A |
| Sodium hydroxide | Sigma-Aldrich | Cat# 221465 |
| TM38837 | MCE | Cat# HY-112340 |
| Ultra High Sensitivity ECL Kit | GlPbio | Cat# GK10008 |
| Deposited data | | |
| GEO data | NIH(Gene Expression Omnibus) | GES14905 |
| GEO data | NIH(Gene Expression Omnibus) | GSE40033 |
| GEO data | NIH(Gene Expression Omnibus) | GSE141804 |
| GEO data | NIH(Gene Expression Omnibus) | GSE106992 |
| Critical commercial assays | | |
| biotin-CTP | Enzo Life Sciences | Cat# ENZ-42818 |
| Click-iT Nascent RNA Capture Kit | Invitrogen™ | Cat# C10365 |
| Dynabeads™ M-280 Streptavidin | Invitrogen™ | Cat# 11206D |
| EZ™ MEGA T7 Transcription Kit | enzynomics | Cat# EZ039S |
| IL-1β mouse uncoated ELISA kit with plates | Invitrogen™ | Cat# 88-7013-76 |
| IL-17A mouse uncoated ELISA kit with plates | Invitrogen™ | Cat# 88-7371-86 |
| IL-23 mouse uncoated ELISA kit with plates | Invitrogen™ | Cat# 88-7230-86 |
| Lipofectamine 2000  Transfection Reagent | Invitrogen™ | Cat# 11668019 |
| NucBlue™ Fixed Cell ReadyProbes™ Reagent (DAPI) | Invitrogen™ | Cat# R37606 |
| Protein A Agarose | Invitrogen™ | Cat# 15918-014 |
| ReverTra Ace® qPCR RT Kit | TOYOBO | Cat# FSQ-101 |
| SensiFAST™ SYBR Hi-ROX kit | Meridian Bioscience, Inc. | Cat# BIO-92020 |
| TNFa mouse uncoated ELISA kit with plates | Invitrogen™ | Cat# 88-7324-76 |
| VECTASTAIN ABC  HRP kit (peroxidase, Rabbit IgG) | VectorLabs | Cat# PK-4001 |
| Experimental models: Cell lines | | |
| HEK 293T | ATCC | Cat# CRL-3216;  RRID: CVCL_0063 |
| Mouse: Bone marrow-derived dendritic cells (BMDCs) | This paper | N/A |
| Mouse: Bone marrow-derived macrophages (BMDMs) | This paper | N/A |
| Raw 264.7 | ATCC | Cat# TIB-71; RRID: CVCL_0493 |
| Experimental models: Organisms/strains | | |
| Mouse: B6.C-Tg(CMV cre)1Cgn (*Cnr1^-/-)^* | The Jackson Laboratory | Cat# Jax 006054;  RRID: MMRRC_071951-JAX |
| Mouse: B6.129P2- Lyz2^tm1(cre)lfo^/J (M-*Cnr1^-/-^)* | The Jackson Laboratory | Cat# Jax 004781;  RRID: IMSR_JAX:004781 |
| Mouse: B6.129S6-Nlrp 3/J (NLRP3^-/-^) | The Jackson Laboratory | Cat# Jax 021302;  RRID: IMSR_JAX:021302 |
| Oligonucleotides | | |
| Primers for qRT-PCR analysis | This paper | Table S2 |
| shRNAs/sgRNAs/siRNA | This paper | Table S3 |
| Recombinant DNA | | |
| pEGFP-C1 | BD Biosciences | Cat# 6084-1 |
| pEGFP-C1-*Nlrp3* 3′UTR AU | This paper | N/A |
| Software and algorithms | | |
| Bedtools (version 2.30.0) | Quinlan and Hall, 2010 | <https://bedtools.readthedocs.io/> |
| Bowtie2 (version 2.4.4) | Langmead and Salzberg, 2012 | <https://bowtie-bio.sourceforge.net/bowtie2/manual.shtml> |
| Cytoscape (version 3.9.1) | Cytoscape Consortium | <https://cytoscape.org/> |
| DAVID | Huang da et al., 2009 | <https://david.ncifcrf.gov/home.jsp> |
| DESeq2 (version 1.32.0) | Love et al., 2014 | <https://bioconductor.org/packages/release/bioc/html/DESeq2.html> |
| edgeR (version 3.36.0) | Robinson et al., 2010 | <https://bioconductor.org/packages/release/bioc/html/edgeR.html> |
| GraphPad Prism 8 (version 8.4.3) | GraphPad Software | <https://www.graphpad.com/> |
| GSEA (version 4.1.0) | Subramanian et al., 2005 | <https://www.gsea-msigdb.org/gsea/index.jsp> |
| IGV (version 2.8.7) | Robinson et al., 2011 | <https://software.broadinstitute.org/software/igv/> |
| Image J | NIH | https://imagej.nih.gov/ij/ |
| Image Lab Software | Bio-Rad Laboratories | Cat#12012931 |
| Python (version 3.9.13) | Python Software Foundation | <https://www.python.org/> |
| R (version 4.1.0) | The R foundation | <https://cran.r-project.org/> |
| RSEM (version 1.3.3) | Li and Dewey, 2011 | <https://github.com/deweylab/RSEM> |
| REVIGO | Supek et al., 2011 | <http://revigo.irb.hr/> |
| Samtools (version 1.15.1) | Danecek et al., 2021 | <http://www.htslib.org/> |
| STAR (version 2.7.1a) | Dobin et al., 2013 | <https://github.com/alexdobin/STAR> |
| TrimGalore (version 0.6.6) | Felix Kruger | <https://github.com/FelixKrueger/TrimGalore> |
| Virus strain | | |
| Ad-CMV-Null | Vector Biolabs | Cat# 1300 |
| Ad-CMV-h-HNRNPD | Vector Biolabs | Cat# ADV-211371 |
| Others | | |
| 0.25% (1X), with porcine trypsin | HyClone | Cat# SH30042.01 |
| 4% paraformaldehyde | BIOSESANG | Cat# PC2031 |
| 30-kDa Amicon centrifugal filters | EMD Millipore | Cat# UFC5030 |
| Biotin-CTP | Enzo Life Sciences | Cat# ENZ-42818 |
| Bradford assay solution | Bio-Rad | Cat# BR500-0002 |
| BSA | Bovogen | Cat# BSA0.1 |
| Castor oil, Ethoxylated | Sigma-Aldrich | Cat# C5135 |
| Cell strainer, 40 μm pore | SPL Life Sciences | Cat# 93040 |
| Cell strainer, 70 μm pore | SPL Life Sciences | Cat# 93070 |
| Chloroform | Sigma-Aldrich | Cat# C2432 |
| Chloroform-d | ZEOtope® | Cat# 300588 |
| DAB substrate kit | VectorLabs | Cat# SK4100 |
| DMF | TCI | Cat# D0939 |
| DNase I | Thermo Scientific | Cat# EN0521 |
| DMEM high glucose (4.5g/l), with L-Glutamine with Sodium Pyruvate | Capricorn Scientific | Cat# DMEM-HPA |
| Dimethyl sulfoxide molecular (DMSO) | Sigma-Aldrich | Cat# D8418 |
| DMSO-d_6_ | ZEOtope® | Cat# 301765 |
| Dulbecco's PBS(1x),w/o ca&mg, w/o phenol red, sterile solution | CELLNEST | Cat# CND200-0500 |
| Dynabeads™ M-280 Streptavidin | Invitrogen™ | Cat# 11206D |
| EDTA | Sigma-Aldrich | Cat# E9884 |
| Eosin Y 1% alcoholic | EMS | Cat# 26051-11 |
| Ethanol | DUKSAN | Cat# 64-17-5 |
| Ethyl Acetoacetate | DAEJUNG | Cat# 7757-82-6 |
| GlycoBlue | Ambion | Cat# AM9515 |
| Hank’s/ balanced salt solution (HBSS) | Gibco | Cat# 14025-092 |
| Hexane | DAEJUNG | Cat# 827 |
| Liberase TL | Sigma-Aldrich | Cat# 5401020001 |
| Master mix | TOYOBO | Cat# TOFSQ-201 |
| Mayer’s Hematoxylin | EMS | Cat# 26602-02 |
| Methanol | DUKSAN | Cat# LH02 |
| Methylcyclohexane | Sigma-Aldrich | Cat# 300306 |
| Methylene Chloride | DUKSAN | Cat# 573 |
| Penicilin/steptomycin (100X) | HyClone | Cat# PS-B |
| Phosphatase inhibitor | Sigma-Aldrich | Cat# P5726 |
| Protease inhibitor | Sigma-Aldrich | Cat# QTPPI1015 |
| PVDF membrane | Bio-Rad | Cat# BR1620177 |
| Protein A Agarose | Invitrogen™ | Cat# 15918-014 |
| Proteinase K | Thermo Scientific | Cat# EO0491 |
| RiboLock RNase Inhibitor | Thermo Scientific | Cat# EO0381 |
| RNAiso plus | TaKaRa bio | Cat# 9109 |
| RPMI 1640, with L-Glutamine | Capricorn Scientific | Cat# RPMI-A |
| Red blood cell lysis buffer (1X) | eBioscience | Cat# 00-4333-57 |
| TEMED | Sigma-Aldrich | Cat# T9281 |
| Tetrahydrofuran | Sigma-Aldrich | Cat# 360589 |
| T-PER^TM^, Tissue protein  extraction reagent | Invitrogen™ | Cat# 78510 |
| TRI reagent | MRC | Cat# TR118 |
| Triton X-100 | Sigma-Aldrich | Cat# T9284 |
| Tween 20 | Sigma-Aldrich | Cay# P2287 |
| Xylene 98.5%, Extra Pure | DUKSAN | Cat# 115 |

**Supplemental Table S2.** Clinical characteristics of healthy and psoriatic patients from whom the skin autopsy samples were collected.

|  | Age | Sex | PASI score | Location |
| --- | --- | --- | --- | --- |
| Healthy control | 37 | F | N/A | neck |
|  | 18 | F | N/A | trunk |
|  | 24 | F | N/A | leg |
|  | 53 | M | NA | arm |
|  | 46 | F | NA | trunk |
|  | 28 | M | NA | trunk |
| Patient | 38 | M | 7.5 | neck |
|  | 45 | F | 17.2 | leg |
|  | 20 | F | 14.3 | trunk |
|  | 55 | M | 19.2 | leg |
|  | 14 | F | 17.6 | trunk |
|  | 27 | M | 33.3 | trunk |

**Supplemental Table S3.** Primer sequencesfor qRT-PCR.

| **Gene** | **Forward primer (5'-3')** | **Reverse primer (5'-3')** |
| --- | --- | --- |
| GAPDH | TGC ACC ACC AAC TGC TTA GC | GGC ATG GAC TGT GGT CAT GAG |
| IL-12p40 | AAC CCT GAC CAT CCA AGT CAA A | CTT GGC CTC GCA TCT TAG AAA G |
| IL-23p19 | GCT TAC AAA CTC GGT GAA CAA CT | GTG GAA TCT CTG CCC ACT TCC |
| TNF-α | GTC TCA GCC TCT TCT CAT TCC TG | TCC TCC ACT TGG TGG TTT GCT AC |
| IL-6 | CCA CTC ACC TCT TCA GAA CG | CAT CTT TGG AAG GTT CAG GTT G |
| IL-1β | TTC GAC ACA TGG GAT AAC GAG G | TTT TTG CTG TGA GTC CCG GAG |
| S100A9 | GCT GGA ACG CAA CAT AGA GAC | TCG CAC CAG CTC TTT GAA TT |
| CB1R | AAG TCG ATC TTA GAC GGC CTT | TCC TAA TTT GGA TGC CAT GTC TC |
| NLRP3 | ATT ACC CGC CCG AGA AAG G | CAT GAG TGT GGC TAG ATC CAA |
| β-defensin3 | GCA TTG GCA ACA CTC GTC AGA | CGG GAT CTT GGT CTT CTC TA |
| AUF1 | AGT CGG AGA GTG TAG ATA AGG TC | GGC CCT TTT AGG ATC AAT GAC TT |
| Caspase-1 | GAG ATG GTG AAA GAG GTG AA | GTG TTG AAG AGC AGA AAG CA |
| CD68 | CCT TAT GGA CAG CTT ACC TTT GG | CTG AGC AGC CTG TAG CCT TAG AG |
| CD86 | TGT TTC CGT GGA GAC GCA AG | TTG AGC CTT TGT AAA TGG GCA |
| Nlrp3 3’UTR (BPD) | CCA AGC TTC TAA TAC GAC TCA CTA TAG GGA GAA AGC AGG ACC ACC AGG T | AGA AAG CAG TAC AAA CAT AC |
| Nlrp3 3U AU R (BPD) | CCA AGC TTC TAA TAC GAC TCA CTA TAG GGA GAG CCT CTC AGC TAC ATT G | AGA AAG CAG TAC AAA CAT ACA A |
| Nlrp3 3U AU R (Cloning) | AAA ACT CGA GTA AGC CTC TCA GCT ACA TTG | AAA AGA ATT CAG AAA GCA GTA CAA ACA TAC |

**Supplemental Table S4.** siRNA sequence for transfection.

| Mouse siAuf1 SC#1 | CCA CAA AGA AAG AUC UGA ATT |
| --- | --- |
| Mouse siAuf1 SC#2 | CCA GUA UUG UAG AGC AAG UTT |
| Human siAUF1 | AAG AUC CUA UCA CAG GGC GAT |
